# Metabolomic and transcriptomic analysis reveals endogenous substrates and metabolic adaptation in rats lacking Abcg2 and Abcb1a transporters

**DOI:** 10.1371/journal.pone.0253852

**Published:** 2021-07-13

**Authors:** Samit Ganguly, David Finkelstein, Timothy I. Shaw, Ryan D. Michalek, Kimberly M. Zorn, Sean Ekins, Kazuto Yasuda, Yu Fukuda, John D. Schuetz, Kamalika Mukherjee, Erin G. Schuetz

**Affiliations:** 1 Cancer & Developmental Biology Track, University of Tennessee Health Science Center, Memphis, Tennessee, United States of America; 2 Department of Computational Biology, St. Jude Children’s Research Hospital, Memphis, Tennessee, United States of America; 3 Metabolon Inc, Durham, North Carolina, United States of America; 4 Collaborations Pharmaceuticals, Inc., Raleigh, North Carolina, United States of America; 5 Department of Pharmaceutical Sciences, St. Jude Children’s Research Hospital, Memphis, Tennessee, United States of America; Universite du Quebec a Montreal, CANADA

## Abstract

Abcg2/Bcrp and Abcb1a/Pgp are xenobiotic efflux transporters limiting substrate permeability in the gastrointestinal system and brain, and increasing renal and hepatic drug clearance. The systemic impact of Bcrp and Pgp ablation on metabolic homeostasis of endogenous substrates is incompletely understood. We performed untargeted metabolomics of cerebrospinal fluid (CSF) and plasma, transcriptomics of brain, liver and kidney from male Sprague Dawley rats (WT) and Bcrp/Pgp double knock-out (dKO) rats, and integrated metabolomic/transcriptomic analysis to identify putative substrates and perturbations in canonical metabolic pathways. A predictive Bayesian machine learning model was used to predict in silico those metabolites with greater substrate-like features for either transporters. The CSF and plasma levels of 169 metabolites, nutrients, signaling molecules, antioxidants and lipids were significantly altered in dKO rats, compared to WT rats. These metabolite changes suggested alterations in histidine, branched chain amino acid, purine and pyrimidine metabolism in the dKO rats. Levels of methylated and sulfated metabolites and some primary bile acids were increased in dKO CSF or plasma. Elevated uric acid levels appeared to be a primary driver of changes in purine and pyrimidine biosynthesis. Alterations in Bcrp/Pgp dKO CSF levels of antioxidants, precursors of neurotransmitters, and uric acid suggests the transporters may contribute to the regulation of a healthy central nervous system in rats. Microbiome-generated metabolites were found to be elevated in dKO rat plasma and CSF. The altered dKO metabolome appeared to cause compensatory transcriptional change in urate biosynthesis and response to lipopolysaccharide in brain, oxidation-reduction processes and response to oxidative stress and porphyrin biosynthesis in kidney, and circadian rhythm genes in liver. These findings present insight into endogenous functions of Bcrp and Pgp, the impact that transporter substrates, inhibitors or polymorphisms may have on metabolism, how transporter inhibition could rewire drug sensitivity indirectly through metabolic changes, and identify functional Bcrp biomarkers.

## Introduction

Breast cancer resistant protein (Bcrp; Abcg2) and P-glycoprotein (Pgp; Abcb1) are multidrug efflux transporters with a substantial overlap in their substrates [1, 2] and a similar tissue distribution [3]. Bcrp and Pgp transport controls substrate (endogenous substances, dietary constituents, and drugs) absorption from the intestine into blood and from the blood into brain and cerebrospinal fluid (CSF). These transporters also enhance substrate clearance from the liver, kidney and other tissues, thereby regulating tissue exposure of their substrates. Thus, the absence or inhibition of these transporters may lead to accumulation of their substrates, leading to toxicity from endogenous compounds or dietary components or altered efficacy of their substrate drugs. These properties make Bcrp and Pgp among the most important FDA-regulated efflux transporters for drug-approval processes [4].

Bcrp and Pgp transporters also efflux endogenous metabolites. Bcrp transports riboflavin (Vitamin B2) [5], uric acid [6], protoporphyrin IX [7] and folic acid [8], while Pgp-mediated transport has been reported for cortisol, aldosterone [9], phospholipids [10], bilirubin [11], endogenous opioid peptides [12] and endocannabinoids [13]. Untargeted metabolomics has been previously used to identify endogenous drug transporter substrates (e.g., for OATP and OAT transporters) [14–16]. Understanding of the transporter’s effect on the metabolome may also help interpret whether metabolomic signatures attributed to drug response, toxicities or side effects (pharmacometabolomics) [17, 18], are revealing a drug’s inhibition/competition with these transporters.

Therefore, by performing untargeted metabolomics, our goal was to understand the effect of both Bcrp’s and Pgp’s absence on the CSF and plasma metabolomes, which could help characterize functional biomarkers of these transporters. In addition, to understand the inter-relationship of transporter substrates with tissue gene expression, transcriptomic analysis was performed on kidney, liver and brain frontal cortex from wild type (WT) and Bcrp-Pgp double knockout (dKO) rats. We compared WT versus dKO male rat CSF and plasma because (a) BCRP shows significant sexual dimorphism (male >female) for expression in many tissues [19]; (b) CSF amount is significantly greater in rats vs. mice; (c) dKO animals have a significantly higher CSF concentration of Bcrp/Pgp dual substrates compared to single Bcrp or Pgp KO animals. This is due to upregulated expression of the alternative transporter (e.g., Bcrp) when the other is deleted (e.g., Pgp) that negates the full impact of each transporter to the disposition of dual substrates [20]; and (d) the CSF-to-plasma ratios of Bcrp and Pgp substrates in KO animals are routinely different from those in WT animals, while the plasma concentration does not always vary between WT and KO animals [21]. Thus, sampling only the plasma or using single KO animals would miss identifying some Bcrp or Pgp substrates.

Our metabolomic analysis results demonstrate that Bcrp and Pgp transporters have a significant and distinct impact on plasma and CSF metabolomes. Moreover, machine learning models identified significantly altered metabolites in dKO animals that are candidate Bcrp and/or Pgp substrates. Biological pathway analysis of the transcriptome of WT vs dKO brain, kidney and liver coupled with gene and metabolite enrichment analysis identified potential important biological functions of Bcrp and Pgp efflux transporters. To our knowledge, this is the first comprehensive effort for complete systemic elucidation of the endogenous biological function of these two important efflux transporters in rat.

## Materials and methods

### Chemicals and reagents

The following chemicals and reagents were utilized for the study: Ketamine (JHP Pharmaceuticals, Rochester, MI, USA); Xylazine (LLOYD Laboratories, Shenandoah, Iowa, USA); RNAlater^™^, TRIzol^™^, Ambion^®^ WT Expression kit, Affymetrix Terminal Labeling Kit and Affymetrix Rat Gene 2.0 St Array (ThermoFisher Scientific, Little Rock, AR, USA).

### Animal experiments and sample collection

Male WT and Bcrp-Pgp dKO Sprague-Dawley rats, 10 weeks of age, obtained from SAGE Laboratories (Saint Louis, MO, USA), were used for the metabolomic/transcritomic study. All animals were provided water and food *ad libitum*. All experimental procedures were approved by the Institutional Animal Care and Use Committee at St. Jude Children’s Research Hospital. A flowchart describing the number of animals in each type of experiment is represented in S1 Fig. Rats were anesthetized with a mixture of ketamine (100 mg/kg) and xylazine (10 mg/kg) and mounted on a stereotaxic apparatus. CSF was collected via the cisterna magna using a scalp vein set (27G X ¾″ T.W.) (Exelint International, Co., LA, CA, USA) and a 100 μL Hamilton syringe (Hamilton, Reno, Nevada, USA). CSF was immediately centrifuged (10,000 x g, 5 minutes) and any RBC pellet discarded. CSF was separated and maintained on dry ice and later stored at -80°C. Any CSF sample with RBC pellet was noted as contaminated, and only samples with no contamination were sent for metabolite analysis. Blood was collected by cardiac puncture into EDTA-coated tubes, centrifuged at 5000 x g for 5 minutes at 4°C, and plasma collected and stored at -80°C. Kidney, liver and frontal cortex of brain were also isolated from each rat. The liver was frozen at -80°C and brain frontal cortex and kidney were stored in RNAlater^™^ following the manufacturer’s instructions until further analysis.

### Metabolomic analysis

Untargeted metabolomics of CSF and plasma samples from WT and dKO rats (n = 8 each genotype) was performed by UPLC-MS/MS and GC-MS-based methods at Metabolon^®^ (Durham, NC, USA) [22]. Metabolon^®^ maintains a metabolite library consisting of retention index (RI), mass/charge (m/z) and chromatographic data of authenticated standard metabolites including the MS/MS spectral information. Named metabolites in the sample were identified by comparing the mass (± 0.005 amu), RI and spectral data of the sample peaks with the library standards, the area under the peak quantified, median normalized and then compared between the WT and dKO groups using the two-sample t-test with Welch’s correction. An estimate of false discovery rate (q-value) was also calculated because of multiple comparisons and q<0.1 was considered to have higher confidence in the data. Significant metabolites were identified based on p< 0.05 and highlighted dark red for increase or dark green for decrease in CSF of dKO compared to WT rats. Trending significant is considered when 0.1>p>0.05 and highlighted light red for increase or light green for decrease in CSF and plasma of dKO compared to WT rats. Principal component analysis was used to identify the data pattern [23] in the samples (WT vs. dKO plasma and WT vs. dKO CSF). Results were further analyzed by Random Forest Analysis to identify important metabolic features that contribute the most towards the group separation. The final output of fold change and associated p and q values were log_2_-transformed, and volcano plots created on Tibco^®^ Spotfire^®^ 7.5 (TIBCO Software Inc., Palo Alto, CA, USA) for better visualization and identification of the significant metabolites. Based on the hierarchical clustering analysis (heatmap) by Metabolon^®^, 3 CSF samples were assumed to be contaminated with plasma, and this information was used to calculate ‘no contamination’ fold change and p-values for the CSF samples, and is listed along with the uncorrected results for all CSF metabolites generated by Metabolon^®^. The non-normalized raw data from Metabolon^®^ was used to calculate the ratio of the signal intensity of metabolites that were detected both in CSF and plasma (CSF/plasma ratio). CSF/plasma ratio of the metabolites were analyzed by two sample t-test considering unequal variance to identify metabolites that have significant alterations in CSF abundance in the dKO rats compared to WT rats. Fold change of (CSF/plasma) dKO / (CSF/plasma) WT were log_2_-transformed and plotted against log_2_ (t values) on Tibco^®^ Spotfire^®^ 7.5 for further identification and better visualization of metabolites with significantly altered CSF abundance in the dKO rats.

### Red blood cell protoporphyrin IX (PPIX) levels

Protoporphyrin IX levels in red blood cells were measured in female WT (n = 10) and Bcrp KO (n = 11) rats by florescence activated cell sorting as described previously [24]. Briefly, peripheral blood was diluted in phosphate-buffered saline and analyzed using a BD LSR II flow cytometer (BD Biosciences, San Jose, CA, USA) at Ex 405 nm and Em 695/40 nm for intracellular PPIX levels. Mean fluorescence intensity of PPIX from 10 to 12 animals in each genotype was analyzed using FlowJo software (Tree Star, Inc., Ashland, OR, USA).

### Metabolomic analysis using MetaboAnalyst

Raw metabolite intensity data from Metabolon^®^ was further analyzed separately using the MetaboAnalyst software (3.0 and 5.0). Briefly, raw data was first screened for missing values and any metabolite with more than 50% missing values was omitted from further analysis. Due to a low number of features (<2000), the data filtration step was skipped. Raw data was further log-transformed and auto-scaled to obtain a Gaussian data distribution for further statistical analysis. Significant metabolites were identified based on ≥1.5- fold increase or decrease in median intensity in dKO compared to WT CSF or plasma, and a p-value less than 0.05. Partial least square discriminant analysis and hierarchical clustering analysis were performed to assess the group separation (WT vs. dKO). The list of significant metabolites was compared with the Metabolon^®^ data to assess the reproducibility of the significant features.

### RNA extraction and microarray analysis

Brain, kidney and liver tissues were weighed, diluted 10 times with TRIzol^™^, homogenized and RNA isolated according to the manufacturer’s instructions. RNA samples were analyzed for quality and integrity on the Agilent 2100 Bioanalyzer and the concentration of RNA was determined using NanoDrop^™^ 8000 spectrophotometer. Tissues from the same five rats/genotype were analyzed by microarray. 125 ng of total RNA was processed for use on the microarray using the Ambion WT kit and the Affymetrix Terminal labeling kit according to the manufacturer’s instructions. The resultant single-stranded cDNA was labeled, fragmented and then hybridized to the Rat Gene 2.0 ST array for sixteen hours at 45°C at 60 rpm. The arrays were then washed, stained, and scanned using the Affymetrix Model 450 Fluidics Station (Affymetric, Inc. Santa Clara, CA, USA) and Affymetrix Model 3000 7G scanner Station (Affymetric, Inc. Santa Clara, CA, USA) using the manufacturer’s recommended protocols. Affymetrix Rat Gene 2.0 ST array was used to perform the gene expression level analysis using Affymetrix Expression Console software and results saved as RMA (Robust Multiarray Averaging) level signal. The transcriptomics data discussed in this manuscript has been deposited in NCBI’s Gene Expression Omnibus (GEO) and are accessible through GEO Series accession number GSE159058.

The RMA signal file was uploaded into Nexus expression 3.0 software (BioDiscovery Inc., El Segundo, CA, USA) along with the biological pathway information for the Affymetrix Rat Gene 2.0 ST Array, pooled based on intensity, corrected for multiple test comparisons at q < 0.05 and fold change ≥1.2, to identify the significantly altered genes and biological pathways.

Gene Set Enrichment Analysis (GSEA) for the transcriptomic data was performed following a previously published method [25].

### Integrated pathway enrichment of metabolite and transcriptome

Combined metabolite and transcript pathways of small molecules were constructed from the SMPDB [26]. Differential analysis of genes and metabolites was performed using LIMMA [27]. Pathway enrichment was performed using Fisher’s Exact Test with Benjamin-Hochberg multiple testing. We used a p-value of 0.1 and FDR of 25% as cutoffs for this analysis.

### *In silico* prediction of BCRP and/or PGP putative substrates or inhibitors

CSF metabolites identified by Metabolon^®^ as significant (p< 0.05) or trending significant (0.05<p< 0.1) were analyzed for their interaction with BCRP and/or PGP by a predictive Bayesian Machine learning model implemented in Assay Central^®^ at Collaborations Pharmaceuticals (Raleigh, NC, USA) according to previously published methods [28–30]. Models were generated for human PGP (ChEMBL TargetID 3467) using 1217 molecules and for human BCRP (ChEMBL5393) with 392 molecules. A five-fold cross-validation was performed to assess the receiver operator characteristic. The metabolites were then scored with these models to output a score as described previously [30]. Furthermore, physicochemical descriptors, e.g. AlogP, molecular polar surface area, hydrogen bond acceptor and donor count, and molecular weight for the metabolites were obtained from the public database. Regression analysis employing standard least square method was used to predict observed fold changes (dKO/WT) in the plasma and CSF for each metabolite using the molecular descriptors, along with the ABCG2 or PGP Bayesian model score to determine a structure-property relationship and understand if the metabolites detected in CSF and plasma have any predictive property of BCRP and/ or PGP substrate.

## Results

### Metabolomics results

#### Metabolomic analysis identifies differences in CSF and plasma metabolite levels between WT and dKO rats

**Fig 1:**
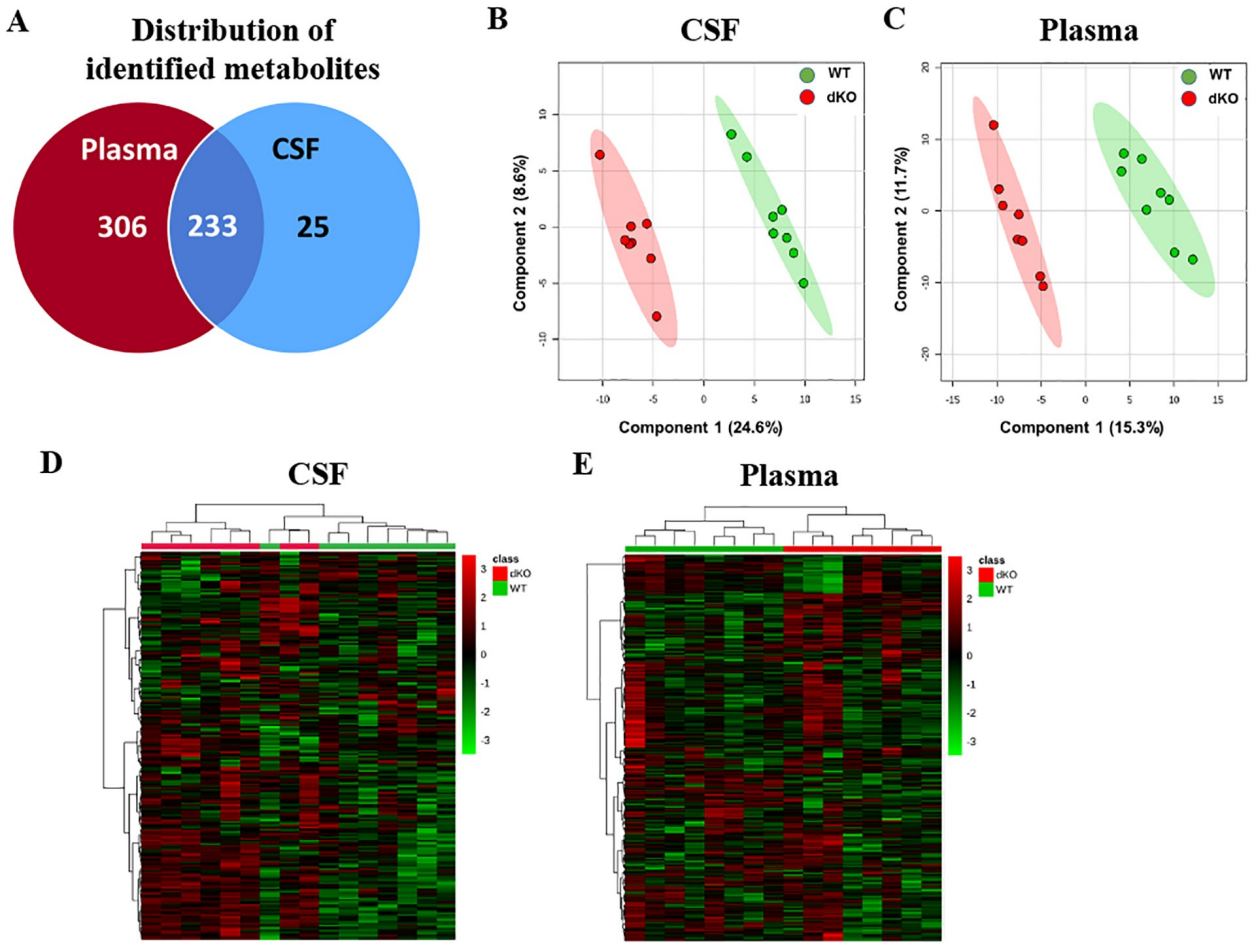
CSF and plasma from WT and Bcrp-Pgp dKO rats have unique metabolite signatures. Venn-diagram showing number distribution of detected named metabolites in CSF and plasma samples (A). PLS-DA analysis 2D-scores plot performed on MetaboAnalyst 3.0, showing distinct group separation between WT and dKO CSF (B) and plasma (C). Hierarchical clustering of the metabolomic data clusters the WT and dKO rat samples together based on their metabolite features in CSF (D) and plasma (E).

The Venn diagram depicts the untargeted metabolomics analysis of metabolites in male Bcrp-Pgp dKO and WT rats that were uniquely present only in plasma (306), or in CSF (25), or in both compartments (233) (Fig 1A). Principal component analysis (PCA) (S2 Fig) and Partial Least Square Discriminant Analysis (PLS-DA) (Fig 1B and 1C) identified unique patterns of metabolites in the CSF and plasma of WT vs. dKO rats. Unsupervised hierarchical clustering analysis of all identified metabolites (Fig 1D and 1E) demonstrated that genotype (WT or dKO) segregated all samples in both tissues.

#### Significantly altered metabolites in CSF and plasma of dKO rats (compared to WT) display unique signatures in each tissue

**Fig 2:**
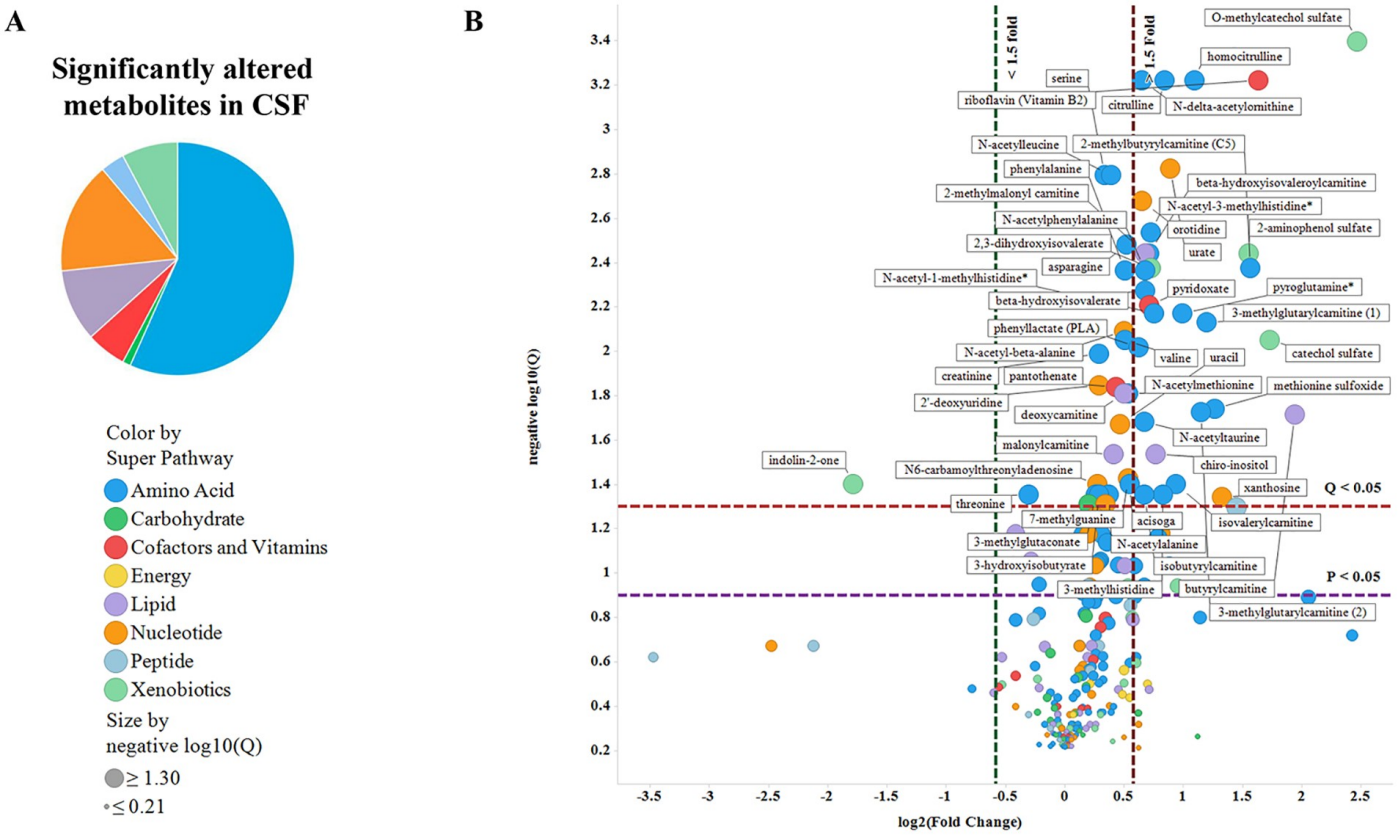
Significantly altered metabolites in CSF of dKO rats (compared to WT) display unique metabolite-class signatures. Pie-chart showing distribution percentages of significant (p<0.05) metabolites in different metabolite classes as designated by the colors in CSF of dKO vs. WT rats (A). Volcano plots (log_10_(q) vs. log_2_(fold change)) plot of CSF metabolites identified by pathway information (color of markers) and their significance (q-value) (size of marker) (B). Data is plotted as log2 (dKO/WT metabolite raw signal intensity) on X-axis vs. negative log (Q-value of metabolite comparison KO vs WT) on Y-axis. Horizontal purple and red lines designate p < 0.05 and q < 0.05, respectively. Significantly altered metabolites (p< 0.05) are identified above the purple line. Similarly, metabolites with 1.5-fold increase or decrease are identified with vertical red and green lines, respectively. Metabolites on the right side of red line or on the left side of green line and above the horizontal purple line are significantly increased (>1.5 fold) or decreased (<1.5 fold) in CSF of dKO vs. WT rats.

**Fig 3:**
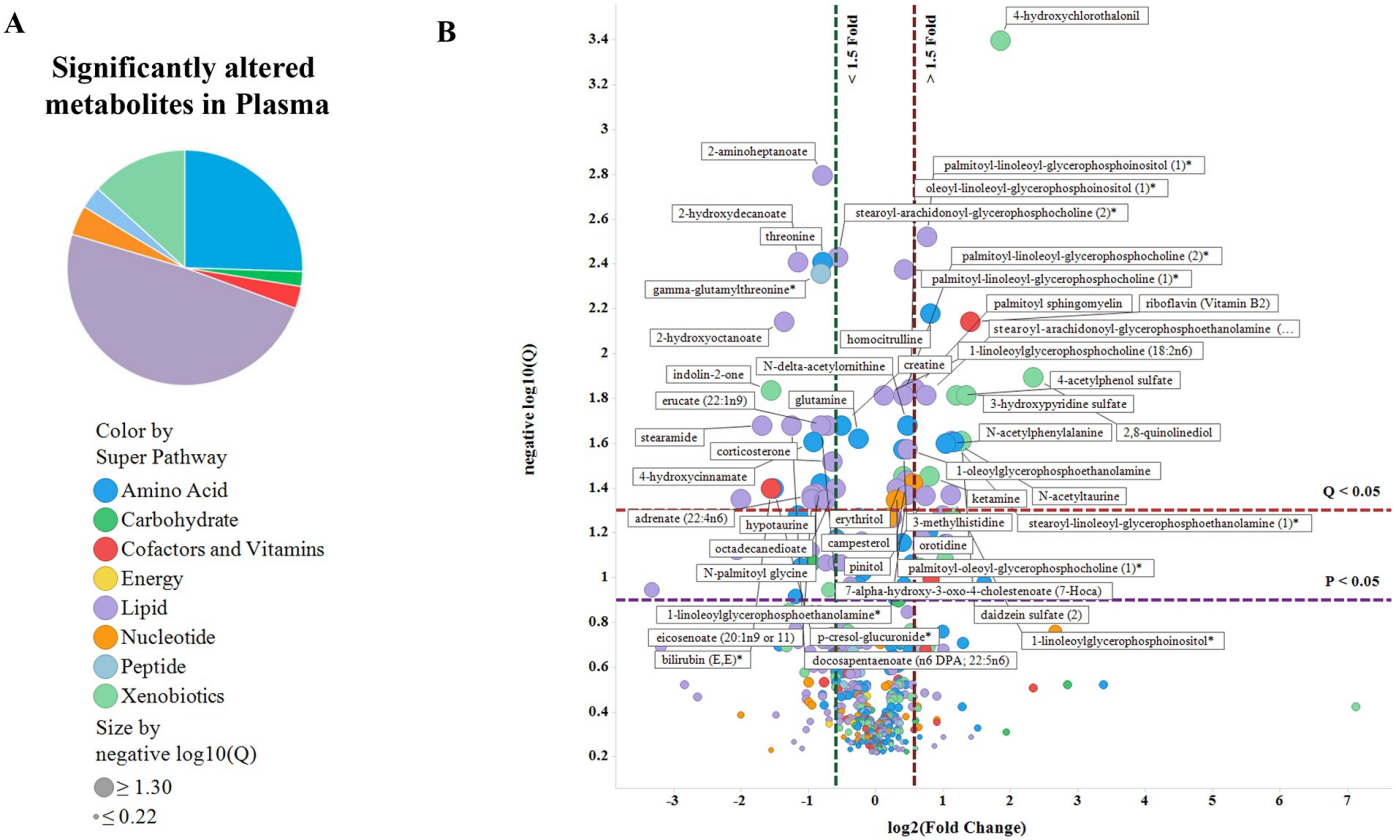
Significantly altered metabolites in plasma of dKO rats (compared to WT) display unique metabolite-class signatures. Plasma metabolite data was analyzed and displayed as shown in Fig 2.

Among the metabolites significantly altered only in the CSF (71), plasma (79), or both (19), amino acid related metabolites were the predominant CSF-altered metabolites (57%) (Fig 2A), while lipids comprised the foremost (49%) significantly altered metabolites in the plasma (Fig 3A) in dKO compared to WT rats. Volcano plots showed that while the majority of metabolites in CSF were increased in dKO rats (Fig 2B), an equal number of metabolites were increased as were decreased in the dKO plasma (Fig 3B). The metabolite with the largest increase in dKO rat CSF was O-methyl catechol sulfate (5.5-fold) (microbiome-generated [31]) and in dKO plasma was 4-hydroxychlorothalonil (3.6-fold) (a soil degradation product of the organochlorine pesticide chlorothalonil [32]). Only one metabolite, indolin-2-one, was significantly decreased (0.29-fold) in the dKO CSF, while many lipid metabolites were significantly decreased in dKO plasma.

Random Forest Analysis (RFA) identified the metabolites with the greatest effect on group separation (WT and dKO) in CSF and plasma (S3 Fig). Analysis of the metabolites with MetaboAnalyst, using a more stringent data filtering protocol, identified 29 metabolites altered significantly (fold change ≥ 1.5) in the CSF and 47 metabolites in the plasma (S4 Fig) of dKO rats compared to WT rats. Metabolites that were more abundant in dKO vs. WT rats included known Bcrp substrates riboflavin and urate in the CSF, and riboflavin and pheophorbide A in the plasma (S5 Fig).

#### Endogenous metabolites and metabolic pathways altered in the CSF and plasma of dKO compared to WT rats

**Fig 4:**
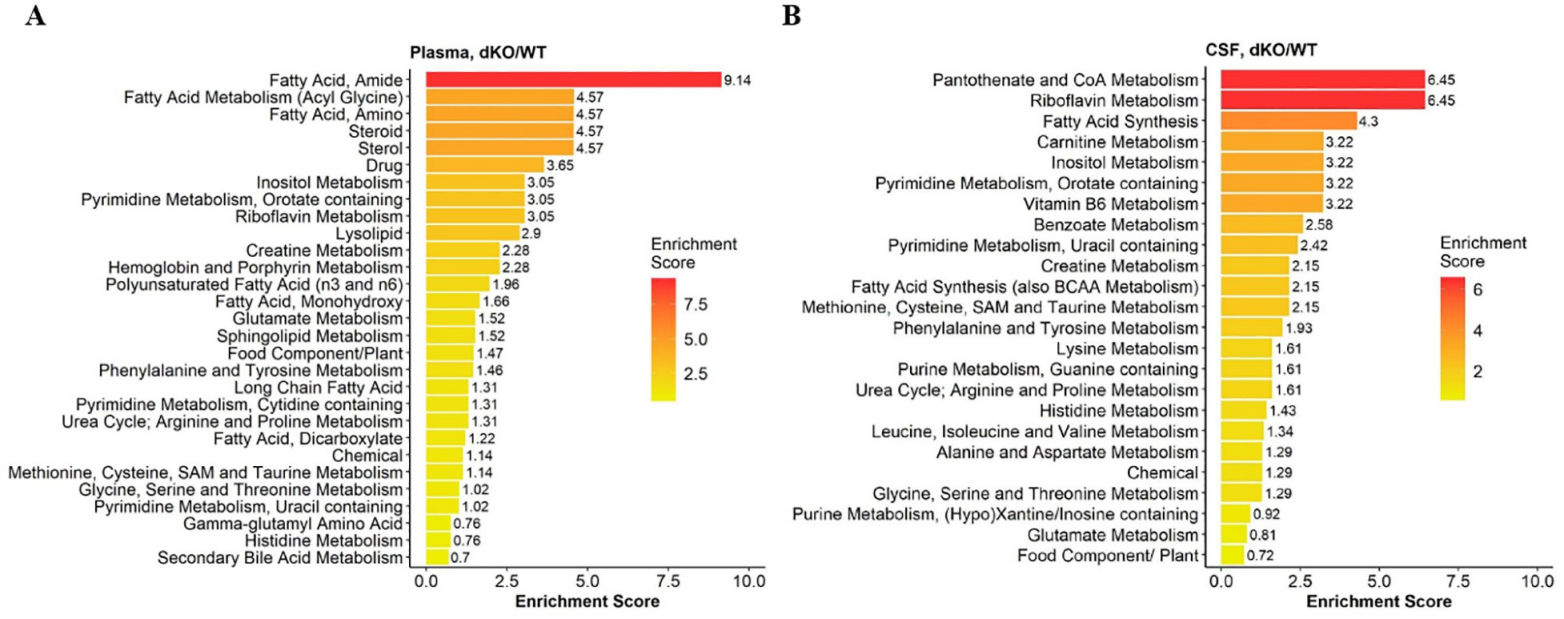
Pathway enrichment and visualization of significant metabolic pathways from Bcrp/Pgp dKO vs WT rat CSF and Plasma metabolites. Significance is calculated at p< 0.01; Rank order is created based on enrichment score: (k/m)/(n/N); k = number of significant metabolites in pathway; m = number of detected metabolites in pathway; n = total number of significant metabolites; N = total number of metabolites.

*Altered plasma lipid metabolism*. The plasma of dKO rats had a significant decrease in medium and long chain fatty acids, unsaturated fatty acids, dicarboxylate, monohydroxy fatty acids, amide and amino fatty acids and polyunsaturated fatty acids (PUFAs) such as eicosenoate, eicosapentaenoate, docosapentaenoate and docosahexaenoate (Table 1A, S1 and S2 Tables), indicating a significant alteration in the fatty acid metabolism pathway. Pathway enrichment analysis of plasma metabolites confirmed that fatty acid pathways were enriched in the plasma (Fig 4).

**Table 1:** A. Decrease in fatty acids in dKO rat plasma. B. Increase in lysolipids in dKO rat plasma. C. Increase in fatty acids in dKO rat CSF.

**A**.			
Sub Pathway	Biochemical Name	**Fold Change**	**p-Value**
Medium Chain Fatty Acid	heptanoate (7:0)	**0.79**	0.08
Medium Chain Fatty Acid	10-undecenoate (11:1n1)	**0.44**	0.05
Medium Chain Fatty Acid	5-dodecenoate (12:1n7)	**0.56**	0.04
Long Chain Fatty Acid	myristoleate (14:1n5)	**0.64**	0.06
Long Chain Fatty Acid	eicosenoate (20:1n9 or 11)	**0.52**	0.01
Long Chain Fatty Acid	erucate (22:1n9)	**0.61**	0.00
Polyunsaturated Fatty Acid (n3 and n6)	docosahexaenoate (DHA; 22:6n3)	**0.59**	0.01
Polyunsaturated Fatty Acid (n3 and n6)	docosatrienoate (22:3n3)	**0.64**	0.07
Polyunsaturated Fatty Acid (n3 and n6)	arachidonate (20:4n6)	**0.75**	0.07
Polyunsaturated Fatty Acid (n3 and n6)	adrenate (22:4n6)	**0.52**	0.01
Polyunsaturated Fatty Acid (n3 and n6)	docosapentaenoate (n6 DPA; 22:5n6)	**0.42**	0.00
Polyunsaturated Fatty Acid (n3 and n6)	mead acid (20:3n9)	**0.51**	0.02
Fatty Acid, Branched	isocaproate	**0.45**	0.07
Fatty Acid, Branched	15-methylpalmitate	**0.76**	0.06
Fatty Acid, Dicarboxylate	azelate (nonanedioate)	**0.76**	0.08
Fatty Acid, Dicarboxylate	tetradecanedioate	**0.6**	0.07
Fatty Acid, Dicarboxylate	hexadecanedioate	**0.6**	0.02
Fatty Acid, Dicarboxylate	octadecanedioate	**0.66**	0.01
Fatty Acid, Dicarboxylate	eicosanodioate	**0.6**	0.01
Fatty Acid, Dicarboxylate	docosadioate	**0.68**	0.01
Fatty Acid, Amide	stearamide	**0.31**	0.00
Fatty Acid, Amide	erucamide	**0.25**	0.01
Fatty Acid, Amino	2-aminoheptanoate	**0.58**	0.00
Fatty Acid, Amino	2-aminooctanoate	**0.65**	0.01
Fatty Acid Metabolism(Acyl Glycine)	N-palmitoyl glycine	**0.54**	0.01
Fatty Acid Metabolism(Acyl Carnitine)	myristoleoylcarnitine*	**0.71**	0.05
Carnitine Metabolism	carnitine	**1.14**	0.03
Fatty Acid, Monohydroxy	2-hydroxyoctanoate	**0.39**	0.00
Fatty Acid, Monohydroxy	2-hydroxydecanoate	**0.45**	0.00
Fatty Acid, Monohydroxy	5-hydroxyhexanoate	**0.67**	0.02
Fatty Acid, Monohydroxy	16-hydroxypalmitate	**0.7**	0.02
Fatty Acid, Monohydroxy	13-HODE + 9-HODE	**0.69**	0.09
Fatty Acid, Dihydroxy	12,13-DiHOME	**0.65**	0.09
Endocannabinoid	oleic ethanolamide	**0.85**	0.07
Inositol Metabolism	pinitol	**1.67**	0.01
Phospholipid Metabolism	glycerophosphorylcholine (GPC)	**1.61**	0.01
Phospholipid Metabolism	glycerophosphoethanolamine	**2**	0.09
Monoacylglycerol	1-oleoylglycerol (1-monoolein)	**0.52**	0.04
Sphingolipid Metabolism	palmitoyl sphingomyelin	**1.49**	0.00
Sterol	7-Hoca	**0.57**	0.00
Sterol	campesterol	**1.38**	0.00
Steroid	corticosterone	**0.64**	0.00
Steroid	11-dehydrocorticosterone	**0.8**	0.07
Primary Bile Acid Metabolism	chenodeoxycholate	**0.51**	0.10
Primary Bile Acid Metabolism	glycochenodeoxycholate	**0.11**	0.09
Secondary Bile Acid Metabolism	deoxycholate	**0.55**	0.06
Secondary Bile Acid Metabolism	glycodeoxycholate	**0.1**	0.03
Secondary Bile Acid Metabolism	taurodeoxycholate	**0.57**	0.06
Secondary Bile Acid Metabolism	tauroursodeoxycholate	**0.58**	0.08
Secondary Bile Acid Metabolism	taurocholenate sulfate	**1.99**	0.01
**B**.			
Sub Pathway	Biochemical Name	**Fold Change**	**p-Value**
Lysolipid	1-stearoylglycerophosphocholine (18:0)	**0.77**	0.03
Lysolipid	2-stearoylglycerophosphocholine*	**0.63**	0.06
Lysolipid	1-linoleoylglycerophosphocholine (18:2n6)	**1.09**	0.00
Lysolipid	1-arachidonoylglycerophosphocholine (20:4n6)*	**0.91**	0.08
Lysolipid	1-oleoylglycerophosphoethanolamine	**1.39**	0.00
Lysolipid	1-linoleoylglycerophosphoethanolamine*	**1.37**	0.01
Lysolipid	1-palmitoylglycerophosphoinositol*	**2.09**	0.01
Lysolipid	1-linoleoylglycerophosphoinositol*	**2.17**	0.01
Lysolipid	oleoyl-linoleoyl-glycerophosphoinositol (1)*	**1.43**	0.00
Lysolipid	palmitoyl-linoleoyl-glycerophosphocholine (1)*	**1.34**	0.00
Lysolipid	palmitoyl-linoleoyl-glycerophosphocholine (2)*	**1.33**	0.00
Lysolipid	palmitoyl-linoleoyl-glycerophosphoinositol (1)*	**1.7**	0.00
Lysolipid	palmitoyl-oleoyl-glycerophosphocholine (1)*	**1.24**	0.01
Lysolipid	stearoyl-arachidonoyl-glycerophosphocholine (2)*	**0.68**	0.00
Lysolipid	oleoyl-linoleoyl-glycerophosphocholine (1)*	**1.18**	0.01
Lysolipid	oleoyl-linoleoyl-glycerophosphocholine (2)*	**1.39**	0.04
Lysolipid	stearoyl-arachidonoyl-glycerophosphoethanolamine (1)*	**1.68**	0.00
Lysolipid	stearoyl-linoleoyl-glycerophosphoethanolamine (1)*	**2.18**	0.00
Lysolipid	2-behenoylglycerophosphocholine*	**0.24**	0.02
Lysolipid	stearoyl-arachidonoyl-glycerophosphoinositol (2)*	**0.87**	0.01

**C**.					
Sub Pathway	Biochemical Name	**Fold Change**	**Fold Change** **(no contamination)**	dKO / WT p-Value	dKO/WT (no contamination) p-Value
Fatty Acid, Dicarboxylate	dimethylmalonic acid	**1.36**	**1.42**	0.03	0.05
Fatty Acid Synthesis	Malonylcarnitine	**1.24**	**1.33**	0.02	0.01
Fatty Acid Synthesis	2-methylmalonyl carnitine	**1.43**	**1.60**	0.00	0.00
Fatty Acid Metabolism (also BCAA Metabolism)	Butyrylcarnitine	**3.77**	**3.84**	0.00	0.00
Carnitine Metabolism	Deoxycarnitine	**1.42**	**1.41**	0.00	0.00
Carnitine Metabolism	Carnitine	**1.18**	**1.14**	0.09	0.18
Ketone Bodies	3-hydroxybutyrate (BHBA)	**0.80**	**0.75**	0.04	0.02
Inositol Metabolism	chiro-inositol	**1.51**	**1.70**	0.02	0.01
Mevalonate Metabolism	3-hydroxy-3-methylglutarate	**0.83**	**0.82**	0.07	0.04
Sterol	7-alpha-hydroxy-3-oxo-4-cholestenoate (7-Hoca)	**0.56**	**0.69**	0.05	0.18

Raw signal intensity of the metabolites was used to compute the fold change (dKO/ WT) and p-value was calculated using Welch’s two sample t-test to identify metabolites as significant (p<0.05) (in dark green & red) or trending significant (0.1>p>0.05) (light green & red). Table 1A lists the significantly decreased overall plasma fatty acid and lipid signature. Significant increase in lysolipids are listed in Table 1B. Increase in few fatty acid and lipid pathway metabolites were observed in CSF (Table 1C).

Conversely, dKO plasma lysolipids increased (Table 1B), which may result from a decline in complex lipid hydrolysis as supported by low levels of glycerol and monoacylglycerols such as 2-palmitoylglycerol and 1-oleoylglycerol. Indeed, high levels of free carnitine in dKO plasma and modestly low levels of long chain carnitine conjugated lipids may highlight a decrease in fatty acid import into the mitochondria, while reduced levels of medium chain fatty acids may further underlie decreased β-oxidation. A similar trend in dKO CSF was suggested by lower levels of the ketone body 3-hydroxybutyrate (Table 1C), a historical marker of lipid oxidation [33, 34].

*Histidine metabolism*. might be altered because 3-methylhistidine, N-acetyl-1-methylhistidine, trans-urocanate and cis-urocanate accumulated in dKO CSF (S3 and S4 Tables), and corresponded with an enriched histidine metabolism pathway in dKO CSF (Fig 4). 1-methylhistidine is a marker of oxidative damage in some tissues and 3-methylhistidine is associated with protein degradation [35]. Importantly, derivatives of histidine with antioxidant properties including carnosine, N-acetylcarnosine, homocarnosine and anserine were diminished in dKO CSF, and this may predispose dKO rats to increased risk of oxidative stress-induced neurological injury as supported by high levels of methionine sulfoxide (oxidation product of methionine) [36].

*Homocitrulline*. The elevated dKO CSF homocitrulline levels (Fig 2) and the homocitrulline/lysine ratio (S3 Table) are likely hallmarks of inflammation secondary to elevated uric acid [37].

*Altered branched chain amino acids (BCAAs) in CSF of dKO rats*. Compared to WT rats, dKO CSF (but not plasma) exhibited elevated levels of the BCAAs isoleucine, leucine and valine, with comparable increases in the level of BCAA degradation products 4-methyl-2-oxopentanoate, isovalerylcarnitine, 2-methylbutyrylcarnitine, tiglylcarnitine and isobutyrylcarnitine (S2 and S4 Tables). The BCAA metabolic pathway was enriched in dKO vs. WT CSF (Fig 4).

**Fig 5:**
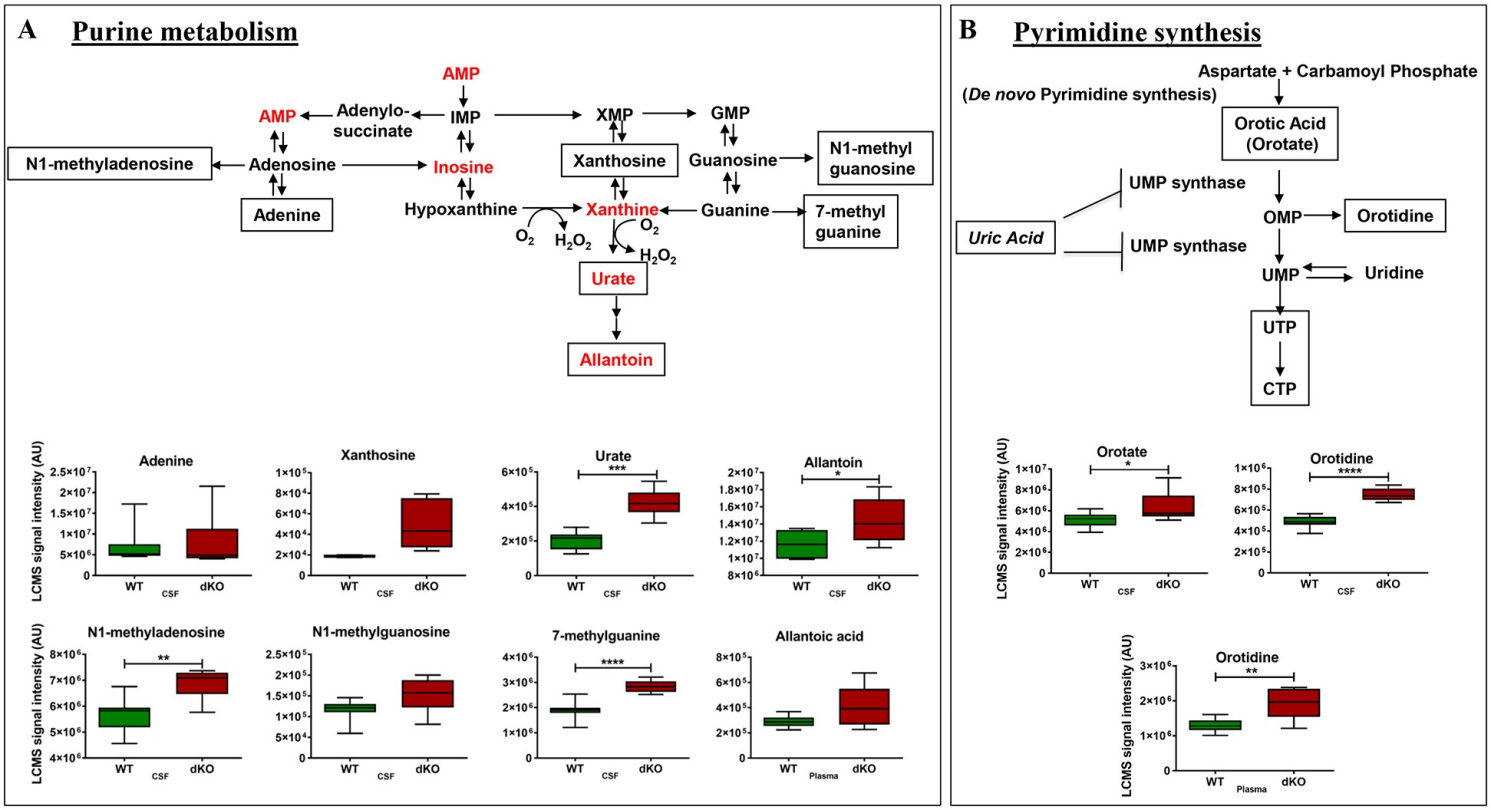
Metabolites in purine and pyrimidine metabolism pathways are altered. Purine metabolites upstream and downstream of urate were increased (indicated by boxed metabolite) in the dKO CSF, and allantoic acid was increased in dKO plasma (A). Two metabolites in pyrimidine synthesis pathway were increased (shown by boxed metabolite) in dKO CSF and plasma (B).

*Altered purine and pyrimidine biosynthesis pathway metabolites*. The purine catabolites xanthosine, urate, allantoin and allantoic acid accumulated in the CSF and plasma of dKO vs. WT rats (Fig 5; S1 and S3 Tables), corresponding to enrichment of the purine metabolism pathway in dKO CSF (Figs 4 and 5). Alteration in purine pathway metabolites could be the direct result of a lack of Bcrp function since Bcrp transports urate and purine analogues [38].

The pyrimidine degradation products uracil, 2-deoxyuridine, pseudouridine and N-acetyl-beta-alanine accumulated in the CSF and plasma of dKO rats and may reflect that they are Bcrp substrates or that there is increased pyrimidine degradation (S1 and S3 Tables). Likewise, the pyrimidine pathway was enriched in dKO CSF (Fig 4). The pyrimidine metabolites orotate and orotidine were also higher in dKO CSF and to a lesser extent in plasma compared to WT rats (Fig 5; S1 and S3 Tables). This might reflect urate inhibition of UMP synthetase blocking the conversion of orotate and orotidine to UMP in the dKO rats [39].

*Altered levels of methylated nucleotides*, *and more generally methylated metabolites*. The metabolites N1-methylguanosine, 7-methylguanosine, N1-methyladenosine and other methylated metabolites including dimethylarginine and S-methylcysteine, 3-methylhistidine, N-acetyl-1-methylhistidine, and S-methylcysteine as well as the methylated metabolite of quercetin (a known Bcrp substrate [40]) were increased in dKO CSF, suggesting Bcrp may recognize some methylated metabolites (S1 and S3 Tables). Alternatively, high levels of these methylated metabolites may suggest a difference in methylation potential between the genotypes that could have potential epigenetic implications (DNA methylation and gene expression).

*Altered bile acids*. The plasma level of some primary bile acids (chenodeoxycholate, glychocholate and glycochenodeoxycholate) (S1 Table) were lower, possibly due to the reduced levels of the precursor 7-alpha-hydroxy-3-oxo-4-cholestenoate (7-HOCA), and decreased bile acid synthesis in dKO animals compared to WT controls.

*Dietary metabolites increased in dKO CSF and plasma*. Included riboflavin and the related biochemical metabolite flavin adenine dinucleotide (FAD), while urate was significantly higher only in dKO rat CSF (S5 Fig; S1 and S3 Tables). The dietary Bcrp substrate pheophorbide A [7] was detected only in the plasma of dKO, but not WT rats (S5 Fig). Another dietary Bcrp substrate, the soy isoflavone daidzein and its metabolite daidzein sulfate, were also significantly increased in the plasma of dKO (2.05- and 2.41-fold, respectively) compared to WT rats (S5 Fig, S1 Table). D-pinitol, a natural compound in plants [41], was significantly increased in dKO rat plasma (Table 1A).

The metabolomic analysis showed significant alteration of gut microbiota metabolites in both CSF and plasma (Table 2). Microbiome metabolites, particularly sulfated metabolites of the amino acid tyrosine, p-cresol sulfate and phenol sulfate [42], decreased 0.45-fold and increased 1.93-fold, respectively, in the CSF of dKO rats, while O-methyl catechol sulfate increased 5.5-fold in the CSF. The microbiome metabolites p-cresol sulfate, equol sulfate, and daidzein sulfate are known Bcrp substrates [43, 44], and given Bcrp’s high affinity for sulfated metabolites, the other microbiota-generated sulfated metabolites might be likely Bcrp substrates as well.

**Table 2:** Gut microbiota metabolites identified in our study.

Metabolites	Fold Change_Plasma	p-Value	Fold change_CSF	p-Value	Reference (PMID)	Exclusive biome?
riboflavin (Vitamin B2)	**2.64**	0.0002	**3.09**	2.00E-07	26283345	
creatine	**0.7**	0.0016	0.95	0.3541	26283345	
4-hydroxycinnamate	**0.53**	0.0023			26861391	
taurocholenate sulfate	**1.99**	0.009			26283345	Yes
p-cresol sulfate	**0.45**	0.0091			26283345	Yes
phenyllactate (PLA)	**1.72**	0.0117	**1.56**	0.0004	27505423	
						
indolelactate	**1.45**	0.0201			26283345	Yes
phenylacetylglutamine	**0.46**	0.0215			26283345	Yes
glycodeoxycholate	**0.1**	0.0309			26283345	Yes
3-(2-hydroxyphenyl)propionate	**0.62**	0.0314			27505423	
phenylacetate	**0.44**	0.0338				
indoleacetate	**0.75**	0.0539	1.53	0.1431	26283345	Yes
deoxycholate	**0.55**	0.0607				
indole-3-carboxylic acid	**0.64**	0.0742				
tauroursodeoxycholate	**0.58**	0.0769			26283345	Yes
phenol sulfate	1.27	0.1189	**1.93**	0.0078	26283345	Yes
Isovalerate	0.86	0.3158	**1.25**	0.0084	26283345	No

Metabolites of gut microbiota origin were identified based on previously published papers as well as microbiome metabolite list maintained at Metabolon^®^. Only significantly (p<0.05) (in dark green & red) and trending significant (0.1>p>0.05) (light green & red) metabolites are listed from both plasma and CSF. Fold change is the ratio of dKO/WT metabolite signal intensity and Welch’s two sample t-test is used to compare WT vs. dKO and calculate significance.

Likewise, there was a significant increase in sulfated metabolites of drugs, chemicals, food components in both plasma (Table 3A) and CSF (Table 3B), that are also likely Bcrp substrates.

**Table 3:** A. Sulfated metabolites significantly altered in the plasma of dKO compared to WT rats. B. Sulfated metabolites significantly altered in the CSF of dKO compared to WT rats.

**A**.			
**Sub Pathway**	**Biochemical Name**	**Fold change**	***p*-value**
Phenylalanine and Tyrosine Metabolism	p-cresol sulfate	**0.45**	0.01
Secondary Bile Acid Metabolism	taurocholenate sulfate	**1.99**	0.01
Benzoate Metabolism	catechol sulfate	**1.4**	0.08
Food Component/Plant	equol sulfate	**1.57**	0.02
Food Component/Plant	4-vinylguaiacol sulfate	**0.41**	0.04
Food Component/Plant	daidzein sulfate (2)	**2.41**	0
Drug	4-acetylphenol sulfate	**2.29**	0
Chemical	sulfate*	**0.87**	0.07
Chemical	2-aminophenol sulfate	**1.44**	0.05
Chemical	3-hydroxypyridine sulfate	**2.53**	0

**B**.					
**Sub Pathway**	**Biochemical Name**	**Fold change** **(no contamination)**	**Fold change**	**dKO / WT** **p-value**	**dKO/WT (no contamination)** **p-value**
Phenylalanine and Tyrosine Metabolism	phenol sulfate	**1.93**	**1.84**	0.01	0.04
Dipeptide	prolylglycine	**1.17**	**1.15**	0.02	0.06
Benzoate Metabolism	catechol sulfate	**3.05**	**3.31**	0.00	0.00
Benzoate Metabolism	O-methylcatechol sulfate	**4.04**	**5.52**	0.00	0.00
Chemical	2-aminophenol sulfate	**2.71**	**2.93**	0.00	0.00

Sulfated metabolites of different pathway origin were also significantly increased or decreased in plasma (Table 3A) and CSF (Table 3B). Raw signal intensity of the metabolites was used to compute the fold change (dKO/ WT) and p-value was calculated using Welch’s two sample t-test to identify metabolites as significant (p<0.05) (in dark green & red) or trending significant (0.1>p>0.05) (light green & red).

The concentrations of a few xenobiotics were altered in the dKO rat plasma (S1 Table). Among those, ketamine, the drug used to anesthetize the rats to obtain CSF (and that we recently published as a putative dual Pgp/Bcrp substrate [45]), was elevated 1.74- and 1.93-fold higher in dKO plasma and CSF, respectively. The amount of 4-hydroxychlorothalonil, a metabolite of the organochlorine pesticide chlorothalonil [32, 46] was elevated 3.6-fold in dKO vs. WT rat plasma.

**Fig 6:**
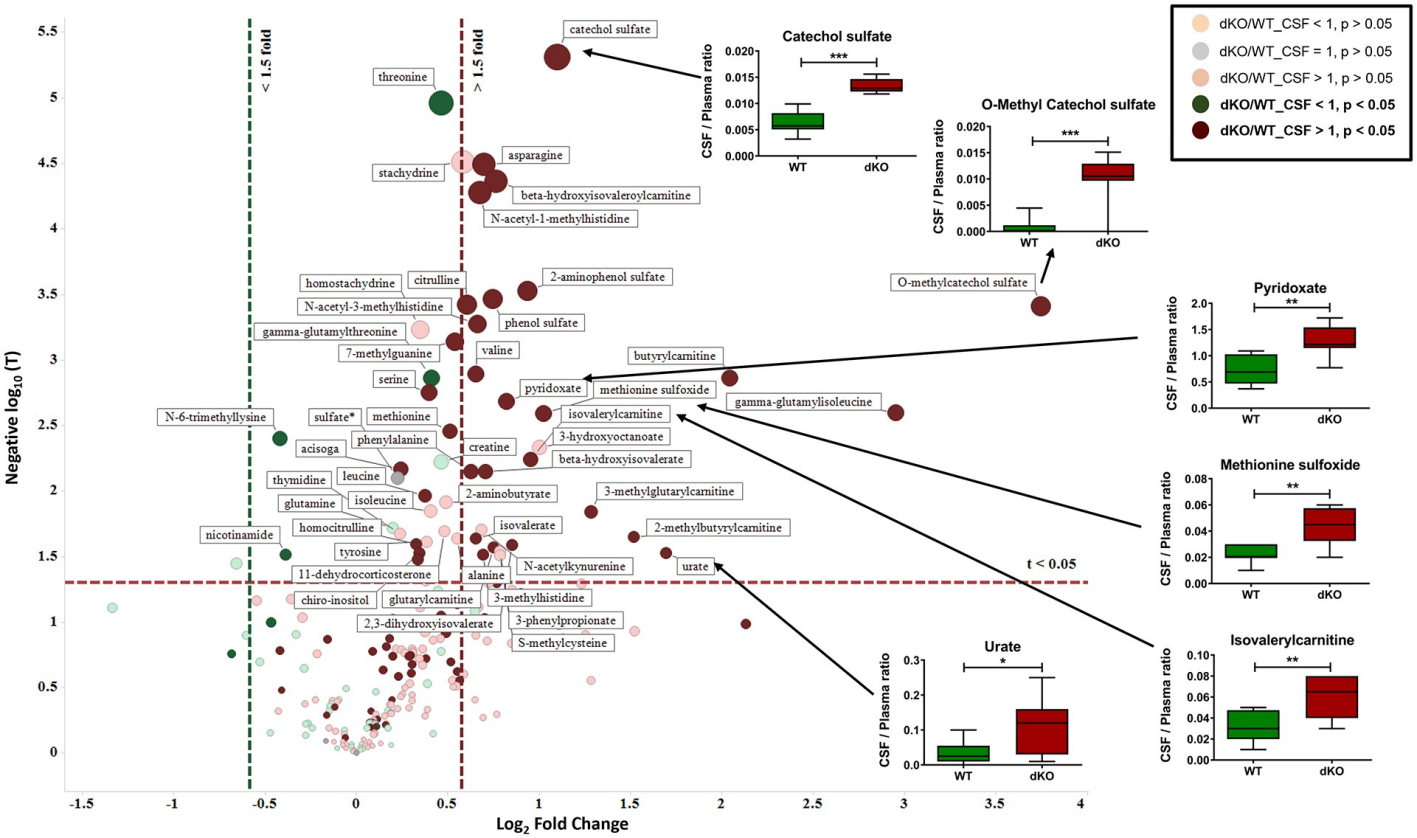
Identification of metabolites with greater accumulation in CSF than in plasma of dKO rats, compared to WT rats. For metabolites identified both in plasma and CSF, a ratio of CSF/plasma metabolite signal intensity was generated for WT and dKO rats. The CSF/plasma ratios of dKO was compared with WT using a two-tailed t-test with two-sample unequal variance. This identified metabolites with significantly higher CSF/plasma ratios in the dKO compared to WT rats at t< 0.05. Log_2_ ratio of CSF/plasma in dKO and WT was also calculated for each metabolite and plotted against negative log_10_ (of their respective t-values) to generate the volcano plot. The horizontal red line describes the t value of 0.05. The vertical red and green lines designate CSF/plasma ratio in dKO that is 1.5 fold higher or lower than WT, respectively. Metabolites that are on the right side of the vertical red line and on top of the horizontal red line, shows metabolites that have significantly higher CSF/plasma ratio in the dKO over the WT rats. Similarly, metabolites on the left of the green vertical line and above the horizontal red line have significantly lower CSF/plasma ratios in the dKO over the WT rats. Dark green and dark red circles represent metabolites significantly high or low, respectively in the CSF/plasma of dKO over WT rats. Box plots indicate known Bcrp substrates and other metabolites that are significantly accumulated in dKO rat CSF.

*Metabolites with greater CSF-to-plasma ratios in dKO vs*. *WT rats*. The CSF-to-plasma ratio of the raw LC-MS/MS signal intensity of metabolites identified in WT and dKO rats were compared because Bcrp and Pgp are known to have a significant impact on the brain permeability of their substrates [47] and many Bcrp and Pgp substrates show an increased CSF-to-brain or CSF-to-plasma ratio in transporter KO mice [21, 48, 49]. Fifty metabolites (Fig 6; S5 Table) had increased CSF-to-plasma ratios in dKO vs. WT rats. Urate, a known Bcrp substrate, had a significantly higher CSF-to-plasma ratio (3.23 fold, p<0.05) in dKO rats, while riboflavin, because it had similar fold increase in both CSF and plasma, had a similar CSF-to-plasma ratio (1.11-fold). Metabolites with significantly increased CSF-to-plasma ratios in dKO vs. WT rats (S5 Table) included catechol sulfate, O-methyl catechol sulfate, methionine sulfoxide, isovalerylcarnitine and pyridoxate. Sixty percent of these metabolites represent amino acid (AA) pathways, with some microbiome-generated AA metabolites (e.g., phenol sulfate from tyrosine and isovalerate [42, 50]). Thirteen percent of the metabolites were xenobiotics, such as catechol sulfate (pyrocatechol sulfate) and O-methyl catechol sulfate, with 2.13- and 13-fold higher CSF-to-plasma ratios, respectively, in the dKO rats, and are likely Bcrp substrates given the preference of Bcrp for sulfated metabolites [51]. Pyrocatechol sulfate, a known uremic toxin, can originate from phenolic compounds in the diet, and be further modified by the gut microbiome, and subsequently sulfated and methylated in the liver [51]. 11-dehydrocorticosterone, a glucocorticoid metabolite, had a CSF-to-plasma ratio of 1.4 in dKO rats, and is a likely Pgp substrate [9].

**Fig 7:**
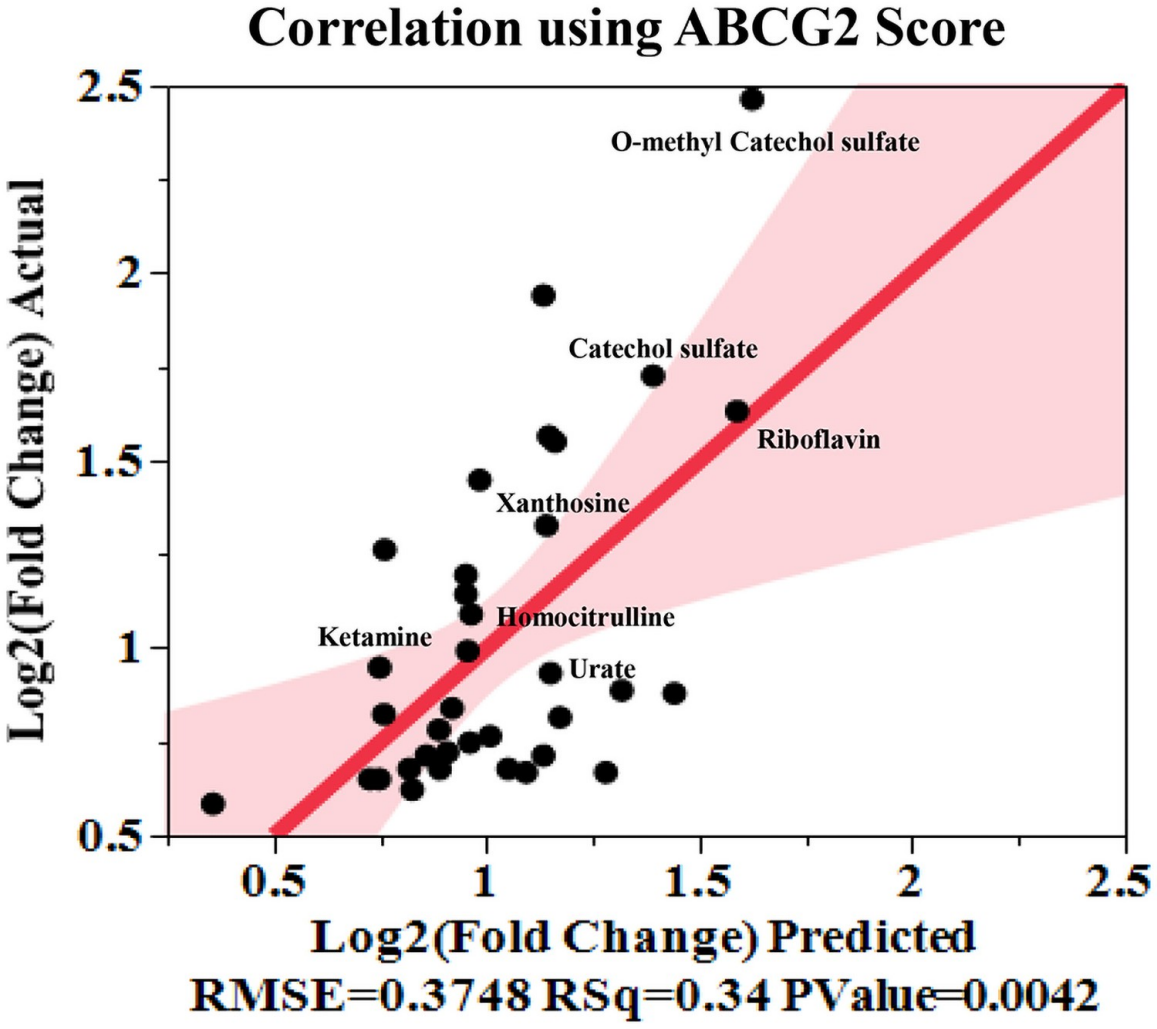
Metabolites with significantly higher abundance in dKO CSF compared to WT rats have structural properties for interaction with Bcrp. Log 2 (fold change) of significantly altered CSF metabolites with > 1.5-fold increase in dKO rats over WT correlated strongly with ABCG2 score, logP and molecular polar surface area of the metabolites.

Since it was unlikely that all 169 metabolites significantly altered in CSF and plasma of dKO rats were transporter substrates, an *in-silico* prediction-based approach was used to determine those metabolites that were most likely Bcrp or Pgp substrates. Bayesian machine-learning models built with compounds known to interact with Bcrp and Pgp were used to score the metabolites based on their level of transporter interaction (high score for higher interaction). Raw data for the significantly altered CSF metabolites that are used for this analysis is presented in S6 Table. Compared to the xenobiotics, many of these metabolites are smaller molecules, and might also have weaker interaction with the transporters, which is also reflected by their lower interaction score. Bcrp or Pgp Bayesian interaction score for each metabolite, along with their molecular descriptors (molecular weight, molecular polar surface area, AlogP etc), were used to predict (see Methods) the observed log_2_-fold change of the metabolites in CSF and plasma. While neither CSF nor plasma metabolites showed a significant correlation with the Pgp score (S6 Fig) CSF metabolites with > 1.5-fold increase in the dKO rats showed a good correlation with the Bcrp score (Fig 7), suggesting many of the scored metabolites are more likely to be Bcrp substrates.

### Transcriptomics results

#### Bcrp and Pgp gene deletion results in an altered transcriptome affecting multiple biological processes in rat kidney, liver and brain

Microarray analysis of WT and dKO rat tissues revealed significant differences in gene expression in kidney (435 genes), liver (398 genes) and brain frontal cortex (240 genes) with 75, 45 and 40 genes commonly altered between kidney and liver, kidney and brain, and brain and liver, respectively. In contrast to a decrease in hepatic *Cyp2b2* reported in Bcrp and Pgp single KO rats [52], hepatic *Cyp2b1* increased in the dKO rats (S7 Table). Similar to findings in transporter single KO rats, expression of none of the major renal drug efflux transporters (S8 Table) was altered between dKO and WT rats. However, some Slc uptake transporters in kidney and liver had altered expression in the dKO rats (S9 Table) (no results were reported for these Slc genes in the transporter single KO rats), e.g. *Slc22a5/OCTN2*, a carnitine transporter, which was significantly decreased in the kidney of dKO rats. However, the loss of Bcrp/Pgp did not result in a change in expression of alternative renal transporters that might have indirectly altered metabolites such as indoxyl sulfate and p-creosol sulfate (Oat1 substrate) [14] or creatinine (Oct1 substrate) [53], strengthening the conclusion that these metabolite changes were caused directly by loss of Bcrp/Pgp. Indeed, indoxyl sulfate has recently been identified as a Bcrp substrate [54].

The altered metabolome in Bcrp/Pgp dKO rats had an apparent physiological impact promoting compensatory transcriptional changes in not only individual genes (S10 and S11 Tables) but also in biological processes in dKO vs. WT rats (Table 4A, S12 Table) including urate biosynthesis and response to lipopolysaccharides in brain, oxidation-reduction processes and response to oxidative stress in kidney, and circadian rhythm genes in liver, with many processes altered in all three tissues in the same direction (Table 4B). Cholesterol and steroid metabolic processes were altered in the kidney and liver of dKO rats, respectively (Table 4A, S10–S12 Tables).

**Table 4:** A. Significantly altered top biological processes based on gene expression changes in dKO compared to WT rats. B. Significantly altered common biological processes between all the tissues.

**A**.					
**Enrichment Term_dKO Brain**	**p-Value**	**Enrichment Term _dKO Kidney**	**p-Value**	**Enrichment Term_dKO Liver**	**p-Value**
retina development in camera-type eye	5.43E-05	response to drug	1.72E-11	aging	3.33E-07
bronchus development	1.43E-04	response to organic cyclic compound	9.79E-09	response to drug	3.06E-06
urate biosynthetic process	1.43E-04	oxidation-reduction process	2.80E-07	wound healing	4.24E-05
response to glucocorticoid stimulus	1.56E-04	Aging	3.26E-07	antigen processing and presentation of exogenous peptide antigen via MHC class II	8.17E-05
Angiogenesis	2.48E-04	positive regulation of apoptotic process	5.23E-07	steroid metabolic process	1.17E-04
retinal cell programmed cell death	4.25E-04	response to organic substance	3.69E-06	glucose 6-phosphate metabolic process	2.29E-04
negative regulation of cell volume	4.25E-04	response to mechanical stimulus	5.86E-06	negative regulation of signal transduction	2.56E-04
trachea formation	4.25E-04	response to food	7.14E-06	response to organic cyclic compound	2.77E-04
vocalization behavior	8.42E-04	response to oxidative stress	8.53E-06	positive regulation of fatty acid beta-oxidation	3.60E-04
negative regulation of actin filament bundle assembly	8.42E-04	response to nutrient	1.31E-05	leukocyte migration involved in inflammatory response	3.60E-04
glomerular visceral epithelial cell development	0.001393	response to cytokine stimulus	1.44E-05	urate metabolic process	3.60E-04
homeostasis of number of cells within a tissue	0.001424	response to glucose stimulus	1.77E-05	maintenance of protein location in cell	3.64E-04
positive regulation of endothelial cell migration	0.001424	Ossification	2.63E-05	cellular protein complex disassembly	3.64E-04
embryonic digit morphogenesis	0.001712	cellular response to cAMP	3.70E-05	response to estrogen stimulus	4.08E-04
response to hypoxia	0.001923	positive regulation of cholesterol efflux	4.02E-05	positive regulation of transcription from RNA polymerase II promoter	5.28E-04
activation of cysteine-type endopeptidase activity involved in apoptotic process by cytochrome c	0.002073	embryo implantation	4.50E-05	circadian rhythm	6.14E-04
visual perception	0.00231	cholesterol homeostasis	5.31E-05	drug metabolic process	7.29E-04
response to lipopolysaccharide	0.002551	negative regulation of ERK1 and ERK2 cascade	5.38E-05	response to glucocorticoid stimulus	7.96E-04
cellular response to interferon-gamma	0.002845	response to vitamin D	6.94E-05	glutathione biosynthetic process	0.001018
glial cell apoptotic process	0.002879	cholesterol metabolic process	9.20E-05	phosphorylated carbohydrate dephosphorylation	0.001078

**B**.			
**Common Biological processes**	**Brain p-Value**	**Liver p-Value**	**Kidney p-Value**
urate biosynthetic process	1.43E-04	0.037835	0.042625
response to glucocorticoid stimulus	1.56E-04	0.000796	0.000459
Angiogenesis	2.48E-04	0.047942	0.002913
response to lipopolysaccharide	0.002551	0.01329	0.003599
circadian rhythm	0.004231	0.000614	0.000171
drug transport	0.004859	0.011985	0.015081
antigen processing and presentation	0.005943	0.02092	0.028569
drug transmembrane transport	0.006026	0.014794	0.018585
antigen processing and presentation of exogenous peptide antigen via MHC class II	0.0102	8.17E-05	0.00013
protein localization in mitochondrion	0.011969	0.0191	0.021544
cellular response to type I interferon	0.011969	0.0191	0.021544
negative regulation of helicase activity	0.011969	0.0191	0.021544
positive regulation of angiogenesis	0.013703	0.003538	0.001348
male gonad development	0.020563	0.006432	0.039839
mitochondrial electron transport; cytochrome c to oxygen	0.023795	0.037835	0.042625
nucleotide-binding oligomerization domain containing 1 signaling pathway	0.023795	0.037835	0.042625
hypoxanthine biosynthetic process	0.023795	0.037835	0.042625
regulation of mitochondrial fission	0.023795	0.037835	0.042625
negative regulation of apoptotic process	0.039936	0.001377	0.001713
brown fat cell differentiation	0.046865	0.017487	0.000351
response to peptide hormone stimulus	0.046952	0.001474	0.000167
intracellular transport of viral proteins in host cell	0.047027	0.002128	0.0027

Top biological processes were identified by enrichment of significantly altered genes in the dKO rat brain frontal cortex, kidney and liver at p < 0.05.

Top 22 common biological processes that were identified by enrichment of significantly altered genes in the dKO rat brain frontal cortex, kidney and liver at p < 0.05.

**Fig 8:**
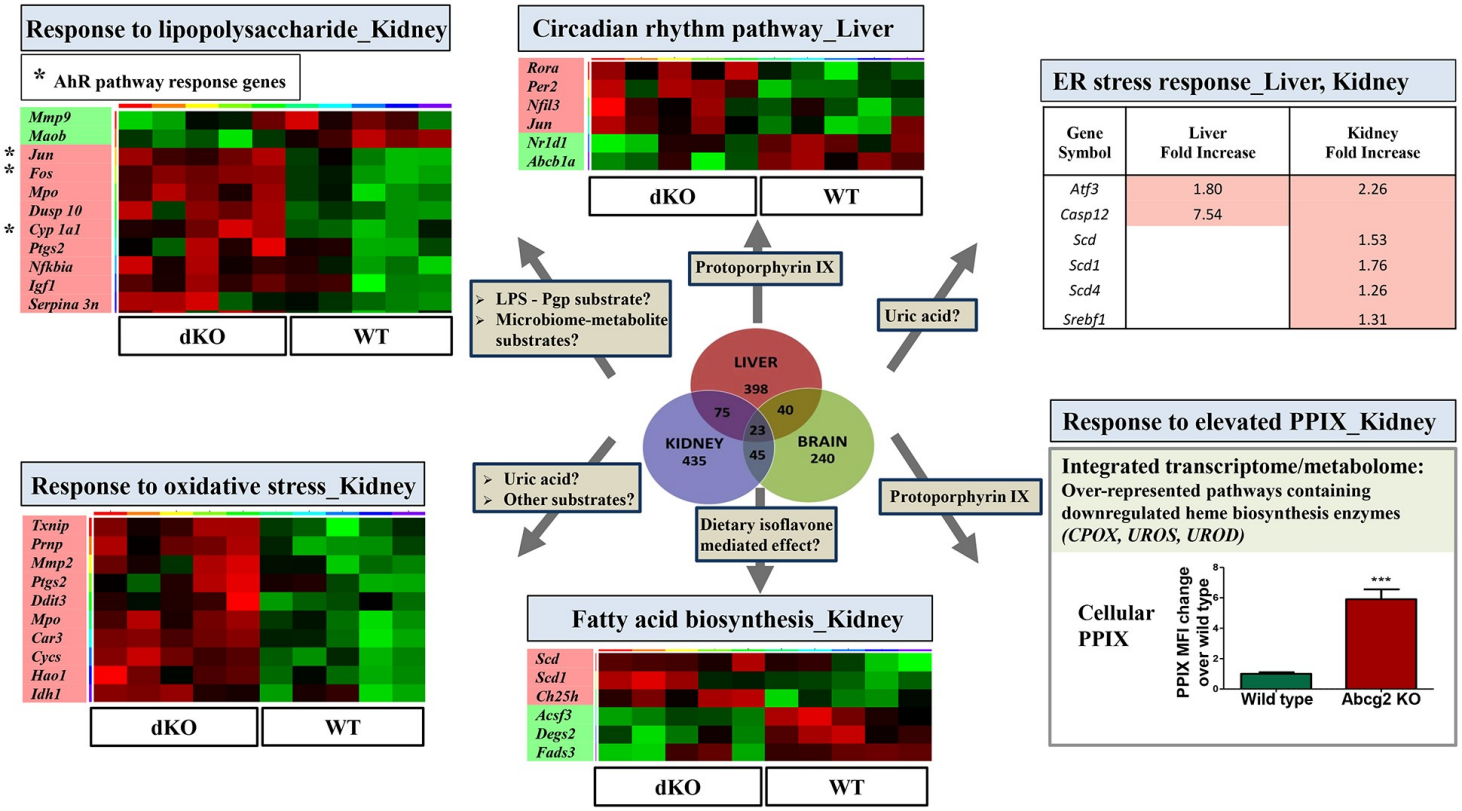
Possible biological pathways altered in the absence of Bcrp and Pgp in the dKO rats, based on both metabolomics and transcriptomic evidence.

*The unfolded protein response/ER stress response appeared to be induced in liver and kidney of dKO rats*. (Fig 8; S10 and S11 Tables). In the absence of Bcrp/Pgp, hepatic genes upregulated included Caspase 12 (*Casp12*), an endoplasmic reticulum (ER) membrane cell death initiator [55]; proteome activator subunit 3 (*Psme3*), a subunit of a proteasome regulator that controls proteasome mediated death; *Fam134b*, involved in degradation of misfolded proteins; and *Marveld1*, a marvel domain containing 1 protein that may regulate decay of damaged mRNA; and in response to those changes, induction of Bri3 binding protein, *Bri3bp*, a protein that in the ER that can decrease apoptosis [56]. Kidneys of dKO rats also showed induction of several proteins in ER stress pathways including activating transcription factor 3, *Atf3*, a transcription factor involved in ER quality control/protein folding after ER stress; *Psme3*; *Nr4a1/Nurr77*, a gene induced by ER stress and that can promote kidney injury [57]; and Uncoupling protein 1, *Ucp1*. It is possible that elevated uric acid is the metabolic cue activating ER stress [58] in Bcrp’s absence, and is also consistent with Bcrp normally protecting from apoptotic injury [59].

*Altered light and odorant sensing signatures in Bcrp/Pgp dKO rats*. A number of genes involved in light sensing and vision were decreased in the dKO brain including retinol binding protein 3 (*Rbp3*) that transports retinoids between photoreceptors and retinal pigment epithelium; G Protein Subunit Alpha Transducin 2 (*Gnat2*) that stimulates coupling of cGMP and rhodopsin for vision; Cone-rod homeobox, (*Crx*) that transcriptionally regulates photoreceptor genes; Phosphodiesterase 6C, (*Pde6c*) important for cone phosphodiesterase activity; phosducin (*Pdc*) that is involved in visual phototransduction; guanylate cyclase activator 1a (*Guca1a*) involved in restoring cGMP for phototransduction [60]; and interphotoreceptor matrix proteoglycan (*Impg1*, *Impg2*) (S10 and S11 Tables). One possible explanation is the elevated protoporphyrin IX-conjugates in the harderian gland, in the absence of Bcrp [61], may improve light sensing/night vision [62], (this is hypothetical, but a similar chemical structure has been shown to improve night vision).

Expression of some vomeronasal receptors (*Vom1r82*, *Vom1r83*) and olfactory receptors (*Olr1668*, *Olr1394*) were altered in dKO kidney (S10 and S11 Tables). Vomeronasal receptors are expressed in human and rodent kidney [63, 64] and over half of olfactory receptors have been identified extra-nasally, including kidney [65]. These receptors can bind directly to metabolites to regulate cell signaling. For example, the olfactory receptor Olfr68 expressed in kidney, binds microbiome-generated short chain fatty acids and regulates blood pressure [66]. Sulfated metabolites of glucocorticoids, bile acids and sex steroids, likely substrates of Bcrp, are potent pheromones known to activate olfactory receptors [67]. In total, these findings suggest that alterations in Bcrp-mediated renal secretion of sulfated chemicals may have a role in influencing signal strength of ligands for renal (and nasal) vomeronasal and olfactory type receptors and their downstream signaling, and perhaps animal behavior.

*Comparison of gene expression between WT and dKO rat tissues by microarray identified ‘circadian rhythm’ pathway as a significantly altered biological pathway*. Bcrp/Pgp dKO liver showed a down regulation of *Nr1d1* (Rev-Erb alpha), a master regulator of the circadian rhythm pathway [68] that is ligand activated by heme, and by high concentrations of protoporphyrin IX (PPIX). While PPIX was below the limits of detection in the plasma of WT and dKO rats, it was elevated (6-fold) in the red blood cells (RBCs) of Bcrp KO rats (Fig 8). It is possible the chronically elevated PPIX levels and chronically activated *Nr1d1* drives the altered rhythmicity of the circadian genes (Fig 8) in dKO vs WT rat transcriptome. RORalpha (*Rorα*), a gene repressed by ligand activated Nr1d1, and *Per2*, a gene downstream of Nr1d1 and RORα regulated *Bmal1*, as well as *NFIL3*, a transcription factor that helps establish circadian rhythm, are out-of-phase between the dKO and WT rats [69] (Fig 8). Interestingly, the International Mouse Phenotyping consortium (IMPC) phenome database showed that mice lacking Bcrp have abnormal sleep behavior/altered percent wake time (www.mousephenotype.org) [70]. The desynchronization of biological rhythms and altered sleeping time would be consistent with symptoms seen in people with porphyrias.

*Integrated plasma metabolome and kidney transcriptome analysis identified the porphyrin pathway as altered in dKO rats*. Integrated pathway enrichment analysis identified down regulation of genes associated with porphyrias and porphyrin metabolism in kidney–specifically, heme biosynthetic enzymes uroporphyrinogen III synthase (*UROS*), uroporphyrinogen decarboxylase (*UROD*) and coproporphyrinogen III oxidase (*CPOX*), and Cytochrome c oxidase assembly protein COX15 homolog (*COX15*) also known as heme A synthase, a gene known to be regulated by porphyrin levels [71] and levels of metabolites in the plasma (S13 Table) and CSF (S14 Table). Presumably, the excess PPIX (Fig 8) in the dKO rats is causing feed-back inhibition of heme biosynthesis. All perturbations (up/down) in mRNAs in liver, kidney and brain and in plasma and CSF metabolites in the same metabolic pathways can be visualized through the hyperlinks in S13 and S14 Tables.

**Fig 9:**
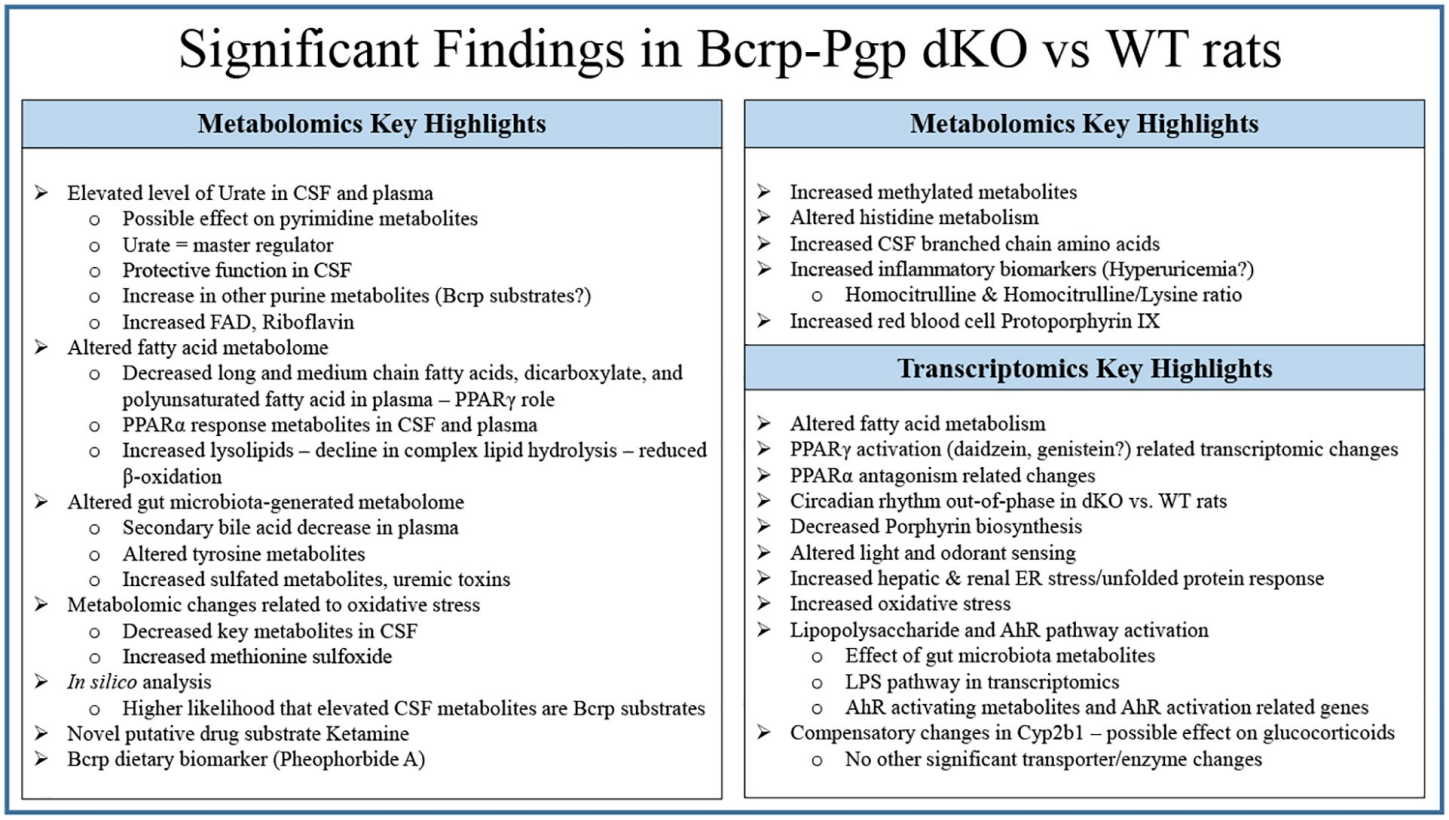
Summary of key metabolomic and transcriptomic findings.

*Use of gene expression and known substrate knowledge for understanding metabolomic features*. Significantly altered biological pathways and the associated gene expression changes indicated that some metabolomic changes may be due to a direct effect of compensatory pathways, or an indirect effect of many metabolites. Fig 8 and S7 Fig show a few potential mechanisms that could underlie the effect of Bcrp/Pgp on gene-metabolite interactions and biological pathways. Key metabolomic and transcriptomic findings from this study are summarized in Fig 9.

## Discussion

The goal of the current study was to examine the effect of Bcrp and Pgp efflux transporter ablation on the rat metabolome and transcriptome in order to better understand their overall endogenous functions, predict drug-induced metabolic alterations/side effects secondary to transporter inhibition by xenobiotics or dietary inhibitors, and predict effects of the well-known BCRP polymorphism [72] on population physiology and drug treatment outcome. In the absence of Bcrp/Pgp, alterations in some metabolites in plasma and CSF might have occurred for reasons such as they were transporter substrates, or represented compensatory transcriptional response to the altered metabolome, and some may result from reprogramming of the gut microbiome by transporter absence.

Collectively, altered CSF metabolites in dKO rats suggest Bcrp and Pgp may contribute to the regulation of a healthy central nervous system in rats. For example, higher CSF uric acid levels would be expected to be beneficial to the dKO rats as uric acid is thought to be protective to the brain, which is sensitive to oxidative damage. Indeed, CSF uric acid levels are inversely correlated with risk of developing Parkinson’s disease, and administration of uric acid is neuroprotective [73–75]. Likewise, citrulline and riboflavin, whose levels were elevated in dKO CSF, are reported to prevent neuronal cell death [76], and protect tissue from oxidative injury [77], respectively. Conversely, diminished dKO CSF levels of the antioxidants carnosine, N-acetylcarnosine, homocarnosine, and anserine could predispose the dKO rats to increased risk of oxidative stress-induced neurological injury as supported by high levels of methionine sulfoxide, a marker of oxidative stress [36]. Additionally, the elevations in CSF (not plasma) BCAA could competitively inhibit transport of aromatic amino acids (tyrosine, tryptophan and phenylalanine) into the brain and consequently may alter synthesis of an array of neurotransmitters. Elevated CSF levels of xanthine, urate, hypoxanthine and glutamate may suggest differences in neuronal function in the dKO rats since their elevated CSF levels are often associated with excitotoxicity in neurological diseases [78]. In addition, CSF has been shown to be important for providing and distributing factors important for stem cell proliferation [79]. For example, purines, known Bcrp substrates, directly stimulate the differentiation of some stem cells/progenitors to differentiated cell lineages [80, 81].

Elevated uric acid in dKO rats may have disparate physiological consequences in different tissues because uric acid is paradoxically both an antioxidant, but also causes oxidative stress [82, 83]. In liver it can cause endoplasmic reticulum stress leading to changes in fatty acid metabolism [58]. Indeed, metabolomic analysis found a significant increase in methionine sulfoxide, a possible biomarker of oxidative stress [36], and ‘oxidative stress’ was a significantly altered biological pathway in the transcriptomic analysis (Table 4A). In pathological conditions, elevated serum urate is paradoxically known to be positively correlated to gout, cardiovascular and renal diseases, while in the CSF it has been shown to be negatively correlated to Alzheimer’s and Parkinson’s diseases [84].

Uric acid appears to be a master regulator of multiple biological systems in dKO rats. Elevated uric acid in the dKO rats is likely leading to the increased CSF pyrimidine anabolites orotidine and orotoic acid (orotate) upstream of UMP synthase, since uric acid is a potent inhibitor of UMP synthase [39] that catalyzes the final two steps in pyrimidine biosynthesis. We propose that elevated uric acid elicits a compensatory decrease in hepatic molybdenum cofactor sulfurase, the enzyme required to sulfurate/activate molybdoflavoprotein requiring enzymes such as xanthine oxidoreductase that converts xanthine to urate. Decreased molybdenum cofactor sulfurase would be expected to decrease xanthine oxidase activity and urate formation [85]. To further identify possible biological responses to elevated uric acid, we investigated for overlap in renal transcription changes in Bcrp/Pgp dKO mice with those of another mouse model with altered uric acid homeostasis—URAT1 KO mice [86]. Carbonic anhydrase 3 (*Car3*) and thyroid hormone responsive SPOT14 protein (*Thrsp*) were two of the most highly induced genes in kidney of both the URAT1 and Bcrp/Pgp dKO mice. Kidney-specific Car3 has been shown to increase in models of proximal tubule dysfunction and oxidative stress [87], suggesting that induction of *Car3*, in the absence of urate transporters, is a biomarker of renal response to elevated uric acid. Additionally, it is possible that the system, sensing the decreased (absent) Bcrp/Pgp, is compensatorily inducing Car3 to try and increase transporter function because another Car isoform has been shown to physically interact with Pgp and increase Pgp ATPase activity and transport activity [88].

The plasma of dKO rats showed a significant decrease in long and medium chain fatty acids, dicarboxylate, and polyunsaturated fatty acids that suggests PPARγ activation. Mice treated with the PPARγ agonist rosiglitazone, have decreased polyunsaturated fatty acids such as mead acid [89] that was also decreased in dKO rat plasma (Table 1A). Corresponding to the decrease in long and medium chain fatty acids, there were increases in choline (GPC) ethanolamine (GPE) and inositol (GPI) glycerophospholipid subclasses (Table 1B) of serum lysolipids in dKO rats. Consistent with apparent PPARγ activation, transcriptomic analysis of dKO vs WT rats showed induction of the PPARγ pathway [89] targets in dKO kidney: uncoupling protein 1 (*Ucp1*, 1.4-fold), fatty acid binding protein 4 (*Fabp4*, 2-fold), leptin (*Lep*, 1.4-fold); and induction in dKO liver and kidney: lipoprotein lipase (*Lpl*, 1.5-fold in liver and 1.3 fold in kidney), and *PPARγ* (1.5-fold in both tissues). Therefore, the metabolomic and transcriptomic results suggest a possible activation of PPARγ in Bcrp/Pgp dKO rats. This seems plausible since dietary isoflavones genistein [90–94] and daidzein [95, 96], known PPARγ agonists, are also Bcrp substrates, and daidzein was increased 2-fold in the plasma of dKO rats (S5 and S7 Figs).

However, our data suggest PPARα antagonism as there was a significant increase of some fatty acids in dKO rat CSF (Table 1C). In support of this proposition, humans treated with the PPARα agonist fenofibrate show decreased urinary acylcarnitine metabolites including propionyl-, isobutyryl-, 2-methylbutyryl- and isovalerylcarnitine [97]. Importantly, some of these metabolites were increased in dKO rat CSF (Fig 2; Table 1C, S3 Table). However, incomplete PPAR-mediated fatty acid oxidation might account for this as acetylcarnitines, are, in part, intermediate products of amino acids (also elevated in dKO CSF) and fatty acids [98], and the levels of many amino acids were elevated in the dKO CSF. The decrease in PPARα target genes in dKO rat liver including interferon-induced protein with tetratricopeptide repeats 1 (*Ifit1*) and carnitine palmitoyltransferase 1a (*Cpt1a*) [97] and the carnitine transporter OCTN2 [99] (S9 Table) is consistent with PPARα antagonism. Further, nicotinamide, a metabolite increased by PPARα activation [97] was decreased in dKO CSF (S3 and S4 Tables). Finally, whereas treatment of mice with the PPARα ligand Wy-14,643 significantly decreased 2,8-dihydroxyquinoline [100], there was a 5-fold increase in 2,8-quinolinediol in the dKO rat plasma (Fig 3, S1 Table). Therefore, the metabolomic and transcriptomic results suggest a possible antagonism of PPARα in Bcrp/Pgp dKO rats presumably due to elevation of some PPARα antagonist/Bcrp/Pgp substrate(s) in dKO rats.

Our findings suggest that the absence of Pgp/Bcrp affects the gut microbiota, that in turn, influences both the plasma and CSF metabolome of dKO rats. Pgp KO mice have been shown to have an altered microbiome [101], and we have evidence that Bcrp’s absence alters the gut microbiome (manuscript in preparation). Evidence for an altered gut microbiome in the Bcrp/Pgp dKO rats includes alterations in metabolites generated exclusively by the microbiome including: (a) decreased secondary bile acids deoxycholate (0.55-fold, 0.1>p>0.05), glycodeoxycholate (0.1 fold, p<0.01), taurodeoxycholate (0.57 fold, 0.1>p>0.05) and tauroursodexycholate (0.58 fold, 0.1>p>0.05), (Tables 1A and 2); (b) altered tyrosine metabolites produced by gut microbiota [102]: cresol sulfate was significantly decreased in the CSF while phenol sulfate was increased in the plasma of dKO rats (Table 2). Phenol sulfate is produced by *E*.*coli* species of bacteria, which have been reported to increase in abundance in mice lacking *Mdr1a* (Pgp) [101, 103]; and (c) increased microbiota-generated metabolites of tryptophan (indolelactate, 1.5-fold), phenylalanine and tyrosine (phenylacetylglutamine, 0.46-fold) in dKO rat plasma and CSF (Table 2). While our findings suggest an alteration in gut microbiome metabolites in the absence of Bcrp/Pgp transporters in rats (Table 2), the extent to which the altered microbiome and/or altered transport of these metabolites individually contributes to their increased levels in the dKO rat plasma and CSF is unknown. This is particularly true for sulfated metabolites whose levels were significantly increased in CSF and plasma of dKO rats (Table 3, S1 and S3 Tables), because many arise from the intestinal microbiome, but may also be Bcrp substrates [51, 104].

The combined metabolomics/transcriptomic findings suggest that microbiome metabolites are activating the lipopolysaccharide (LPS) pathway and the aryl hydrocarbon receptor (AhR) in the Bcrp/Pgp dKO rats (Fig 8). DKO CSF metabolites that are increased are early biomarkers of LPS-induced sepsis in rats [105], specifically palmitoyl carnitine, acetyl-L-carnitine, propionyl-L-carnitine, methionine, N-acetyl methionine and methionine sulfoxide, and branched chain amino acids (Table 1C, S3 Table). In addition, transcriptomic analysis found ‘response to LPS’ as one of the top ten significantly altered biological pathways in liver, kidney and brain of dKO rats (Table 4B). AhR is activated by microbiota-generated tryptophan metabolites/uremic toxins [106] such as indoleacetate and 3-indoxyl sulfate (a Bcrp substrate) [54], and increased levels of at least one of these metabolites in dKO rats could be contributing to ligand activation/Ahr-mediated induction of *Cyp1a1*, *Fos* and *Jun* in kidney and NAD(P)H dehydrogenase quinone 2 (Nqo2), an antioxidant enzyme, in brain (S10 and S11 Tables) [107].

CSF, as opposed to plasma, might be a better matrix to identify Bcrp and/or Pgp endogenous substrates because of the significant role of BBB Bcrp/Pgp on the brain penetration of their substrates [108, 109], and the possibility that alternative transporters in kidney or liver offsets their systemic impact for shared substrates. Indeed, when we used *in silico* Pgp and Bcrp interaction scores generated by a predictive Bayesian Machine learning model, along with molecular descriptors (AlogP, molecular weight, polar surface area) to predict the fold change of metabolites observed in plasma and CSF, only significantly altered metabolites in CSF, and not plasma, displayed a significant correlation between predicted and observed data (Fig 7, S6 Fig).

The metabolomic analysis of Bcrp/Pgp CSF and plasma identified novel exogenous substrates such as ketamine, the drug used for anesthetizing the rats, that was significantly increased in both CSF and plasma of dKO rats and that we recently showed was a putative dual Pgp/Bcrp substrate in mice [45]. Interestingly, there are other studies showing that ketamine, as a receptor antagonist, has diverse effects on endogenous metabolism [110]. Hence, considering ketamine is a fast-acting mitochondrial toxicant, it is possible that the different concentrations of ketamine in dKO vs WT rats might have an impact on the metabolomic changes observed, and that could be investigated in future studies. Our observation that 4-hydroxychlorothalonil, a metabolite of the pesticide chlorothalonil [32, 46] was increased in the plasma of dKO rats, indicates possible elevated exposure risk to this pesticide in people with compromised BCRP and/or PGP function.

A previous study of gene expression changes in Pgp and Bcrp WT vs. single KO rats [52], profiled changes in expression of 112 drug metabolism and drug uptake and efflux transporter genes in the liver, intestine, brain and kidney because the Bcrp and Pgp KO models have historically been used to compare the pharmacokinetics of drug transporter substrates with WT animals. The only significant changes they found [52] were increased intestinal carboxylesterase in Pgp KO and catechol-o-methyltransferase in Bcrp KO tissues, while our study did not analyze intestine gene expression and catechol-o-methyltransferase was decreased (P<0.016) in dKO rats. Similar to findings in Pgp or Bcrp single knockout rats [52], dKO rats did not show significant compensatory changes in expression of renal or hepatic uptake (S9 Table) or efflux transporters (S8 Table). There was a significant increase (2-fold) in hepatic *Cyp2b1* expression in dKO rats (S7 Table), an enzyme that metabolizes glucocorticoids, and this might help explain the decrease in corticosterone in dKO rat plasma (S1 Table, S7 Fig), which might have been expected to increase in animals lacking Pgp [9]. In total, similar to single transporter KO rats [52] dKO rats showed only modest changes in ADME genes suggesting dKO rats could be used for studies of transporter-mediated pharmacokinetics, particularly for dual Pgp/Bcrp substrates.

However, unlike our report, the previous transcriptomic analysis of Pgp and Bcrp single KO rats [52] did not comprehensively analyze changes in other genes. The transcriptomic changes in brain, liver and kidney of dKO vs WT rats not only suggested apparent PPARγ activation and PPARα antagonism, likely in reaction to altered levels of receptor ligands, but also that in response to elevated PPIX, porphyrin biosynthesis decreased and the circadian rhythm is out-of-phase. Intriguingly, there was altered expression of light and odorant sensing genes in the dKO rats perhaps in answer to increased PPIX-conjugates and altered levels of vomeronasal and odorant receptor ligands, respectively. Induction of the unfolded protein response/ER stress response in liver and kidney of dKO rats may have resulted from elevated levels of uric acid, while perturbations in the levels of intestinal microbiota metabolites in dKO rats may have led to activation of the AhR receptor and LPS pathway. In total, these results suggest that Bcrp/Pgp mediated transport of endogenous, xenobiotic and microbiota generated chemicals impacts multiple biological pathways (Table 4A and 4B and Fig 9).

Endogenous drug transporter biomarkers are emerging tools for better predicting drug transport and disposition. Currently, no endogenous probe exclusive for Pgp efflux has been identified [111]. While PPIX is an endogenous Bcrp biomarker, it was apparently restricted to RBCs and not present in the dKO plasma. A number of endogenous metabolites associated with Bcrp function were significantly altered in the dKO rat CSF and/or plasma (e.g., urate, riboflavin). Indeed, a human metabolomics genome wide association study of 7,824 adults [112, 113] found plasma urate levels correlated with BCRP rs2231142, the coding variant associated with decreased BCRP expression [72]. Also, multiple studies have reported that patients who carry ABCG2 rs2231142 are more likely to have gout, and hence, higher serum levels of uric acid [114]. A candidate Bcrp dietary biomarker is pheophorbide A (PhA), a chlorophyll metabolite previously identified as a selective Bcrp substrate [7, 115] that was only detected in Bcrp/Pgp dKO, but not WT rat plasma (S5 Fig, S1 Table), and that we recently identified as a probe which can be used to test for oral Bcrp drug-drug interactions *in vivo* [116]. While no selective Pgp metabolite biomarkers were definitively identified in this study, notably the primary bile acid glycochenodeoxycholate was significantly decreased in the plasma of dKO rats, and its levels in human plasma were negatively associated the ABCB1 SNPs [112, 113]. Although N-acyl-ethanolamine-type endocannabinoids (e.g., oleic ethanolamide), were recently identified as Pgp substrates [13], oleic ethanolamide was only weakly altered (decreased) in dKO rat plasma (Table 1A) demonstrating it would not be a suitable systemic Pgp biomarker.

This systems level understanding of the metabolomic impact and altered biological function due to lack of function of Bcrp and Pgp transporters may lead to better understanding of the desired / undesired effects of therapies that interact with Bcrp and Pgp. For example, pharmacometabolomic studies have identified changes in metabolites following drug exposure. It is possible that some direct effects and side-effects of medications on metabolism [117] result from inhibition of these transporters–i.e., transporter inhibition is causing the metabolomic changes. It will be of interest in the future to compare the metabolic signatures of some drugs profiled in pharmacometabolomics studies with metabolite changes observed in the dKO rats.

Finally, it should also be considered that the absence of ABCG2 function could rewire drug sensitivity indirectly through metabolic changes. For example, since elevated uric acid and subsequently elevated orotate can antagonize 5-fluorouracil metabolism and toxicity [39], it remains to be tested whether persons with ABCG2 genetic variation or inhibition have altered 5-fluorouracil sensitivity. Similarly, elevated levels of histidine and urocanic acid in the absence of ABCG2 function might enhance methotrexate sensitivity in these same individuals. Methotrexate kills cells by inhibiting dihydrofolate reductase and depleting tetrahydrofolate (THF) required for nucleotide synthesis. Elevated histidine and urocanic acid seen in the absence of Abcg2 function would be anticipated to increase metabolism of THF (a co-factor for formimidoyltransferase cyclodeaminase) further depleting THF and nucleotide synthesis and enhance methotrexate cytotoxic effects [118].

Overall, our study highlighted a significant impact of Bcrp and Pgp efflux drug transporters on the systemic metabolome and transcriptome. Further studies are crucial to establish their contribution on selective pathways, but at a systemic level, our study indicated an essential biological role of these transporters. Most importantly, our results suggest that long-term inhibition of these transporters might have significant consequences with respect to neurological function, gut health and immune-inflammatory status of an individual.

## Supporting information

S1 Fig Flow chart of animals used in this study. (TIF)

S2 Fig Metabolites in CSF and plasma are differentially expressed between WT and Bcrp-Pgp dKO rats. Principal Component Analysis of median normalized named metabolites in CSF (A) and plasma (B) identified significant pattern separation between WT and dKO rats. Hierarchical clustering analysis of the fold change (dKO/WT) data in CSF (C) and plasma (D) showed overall separation between WT and dKO rats.
(TIF)

S3 Fig Random forest identifies metabolites that significantly distinguishes between WT and dKO group of mice. Random forest analysis (RFA) was used to calculate group separation and identify metabolites with highest importance for group separation. RFA identified two groups (WT and KO) with 100% accuracy in both CSF (A) and plasma (B). Metabolites were plotted against their Mean Decrease in Accuracy, calculated by RFA. Metabolites have higher importance on group separation as we move up along the Y-axis and they are also color-coded to identify which pathway the metabolites belong to.
(TIF)

S4 Fig Independent analysis of metabolome data with MetaboAnalyst identifies similar metabolites, along with known Bcrp substrates. Raw data from Metabolon were analyzed using MetaboAnalyst 3.0. Significant metabolites were identified based on p < 0.05 and fold change > 1.5. Fold change of all significant metabolites were plotted against their p-value. The red arrow indicates known substrates of Bcrp and/or Pgp.
(TIF)

S5 Fig Known endogenous substrates of Bcrp were increased in abundance in both plasma and CSF of dKO rats. Raw LCMS signal intensities are plotted for each genotype (n = 8), with significance calculated at p < 0.05 by the Mann-Whitney test.
(TIF)

S6 Fig Metabolites with significantly higher abundance in dKO CSF compared to WT rats do not have structural properties for interaction with Pgp. Log 2 (fold change) of significantly altered CSF metabolites with > 1.5 fold increase in dKO rats over WT did not correlate with PGP score, logP and molecular polar surface area of the metabolites (p = 0.1519).
(TIF)

S7 Fig Proposed mechanism for Bcrp/Pgp substrate alterations in dKO rats driving gene expression changes and subsequent compensatory differences in metabolite levels. Proposed mechanism for reduction in corticosterone observed in the dKO rat plasma due to glucocorticoid receptor mediated induction of the Cyp2b1 enzyme in dKO rat liver (observed in microarray analysis of liver tissue) (A). Proposed mechanism for reduction of plasma fatty acids by elevated Bcrp substrates genistein and daidzein activating PPARγ (B).
(TIF)

S1 Table Heat map of statistically significant biochemicals in the plasma of dKO vs WT rats. (XLSX)

S2 Table Comparison of significant metabolites (p< 0.05 and fold change > 1.5, dKO/WT) in rat plasma identified by Metabolon and also by MetaboAnalyst analysis. (XLSX)

S3 Table Heat map of statistically significant biochemicals in the CSF of dKO vs. WT rats. (XLSX)

S4 Table Comparison of significant metabolites (p< 0.05 and fold change > 1.5, dKO/WT) in rat CSF identified by Metabolon and also by MetaboAnalyst analysis. (XLSX)

S5 Table List of metabolites with significantly increased or decreased accumulation in CSF of dKO vs WT rats. (XLSX)

S6 Table List of metabolites and associated in silico parameter values, PGP and BCRP ChEMBL score, fold change in CSF (dKO/WT) observed in metabolomic study. (XLSX)

S7 Table Significantly altered CYP genes in dKO rats compared to WT rats. (XLSX)

S8 Table Significantly altered ABC transporter genes in dKO rats compared to WT rats. (XLSX)

S9 Table Significantly altered SLC transporter genes in dKO rats compared to WT rats. (XLSX)

S10 Table Significantly altered genes identified by the Nexus expression analysis, at p < 0.05 and fold change > 1.2-fold. (XLSX)

S11 Table Results of analysis of microarray data by GSEA (Gene set enrichment analysis). (XLSX)

S12 Table Biological processes perturbed in Bcrp/Pgp dKO vs. WT rat brain frontal cortex, kidney and liver identified by enrichment of significantly altered genes. (XLSX)

S13 Table Metabolic pathways in Bcrp/Pgp dKO vs. WT rats identified by an integrated analysis of kidney transcriptome and plasma metabolome. (XLSX)

S14 Table Biological pathways perturbed in Bcrp/Pgp dKO vs. WT rats identified by an integrated analysis of transcriptomic and metabolomic data. (XLSX)
