# Supplementary figures and images for: Metabolomic and transcriptomic analysis reveals endogenous substrates and metabolic adaptation in rats lacking Abcg2 and Abcb1a transporters
Source: PLoS One. 2021 Jul 13;16(7):e0253852. doi: 10.1371/journal.pone.0253852 (PMC8277073; doi:10.1371/journal.pone.0253852)

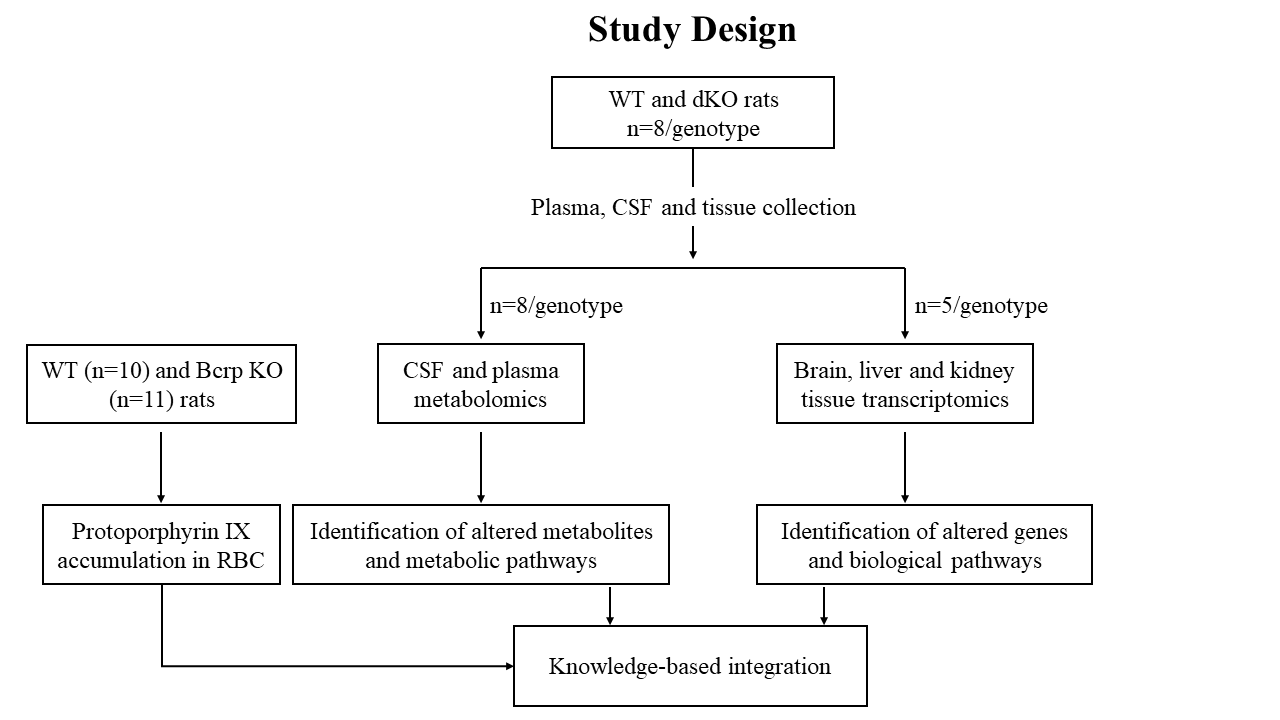

Supplement: S1 Fig — (TIF) [file pone.0253852.s001.tif]

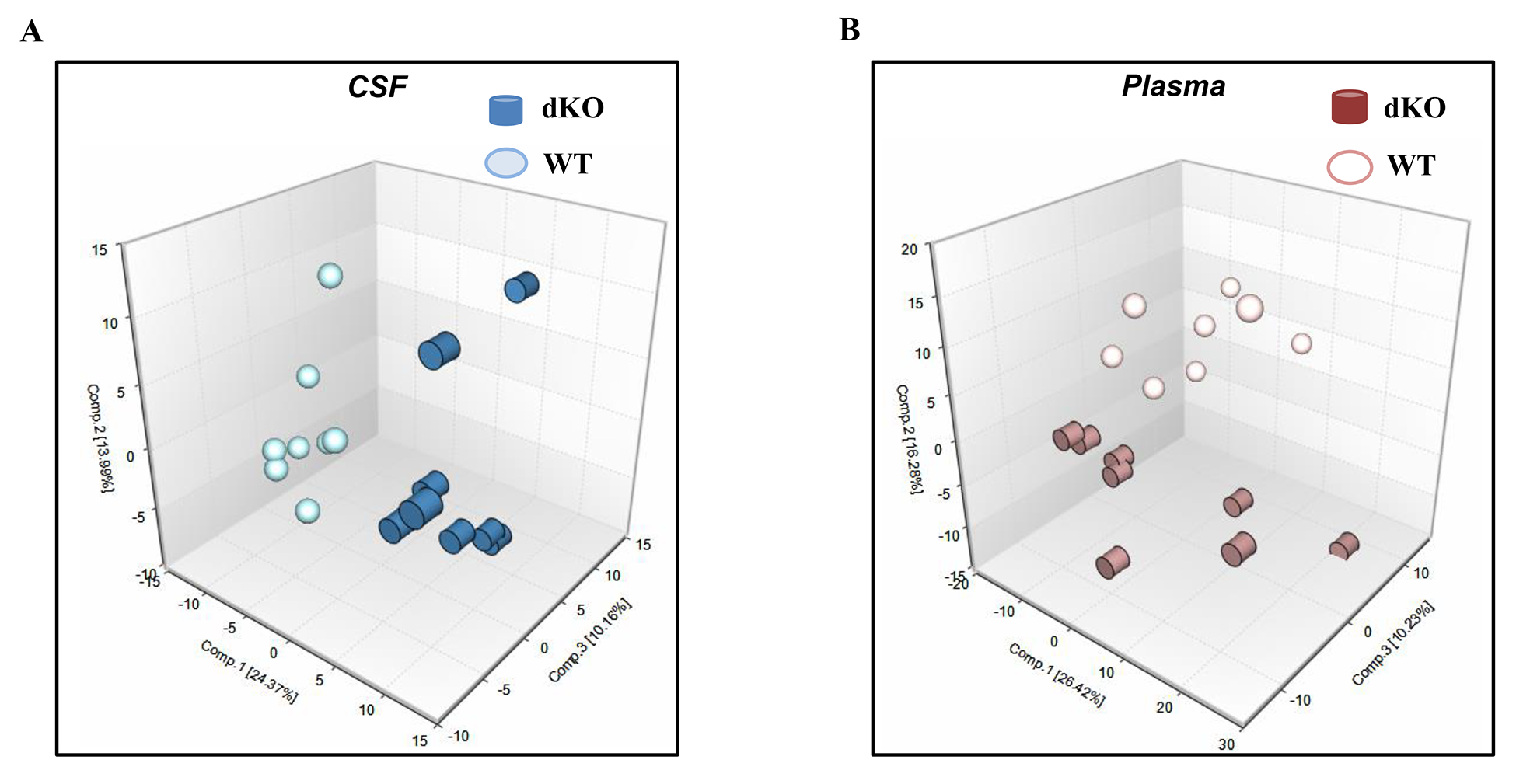

Supplement: S2 Fig — Principal Component Analysis of median normalized named metabolites in CSF (A) and plasma (B) identified significant pattern separation between WT and dKO rats. Hierarchical clustering analysis of the fold change (dKO/WT) data in CSF (C) and plasma (D) showed overall separation between WT and dKO rats. (TIF) [file pone.0253852.s002.tif]

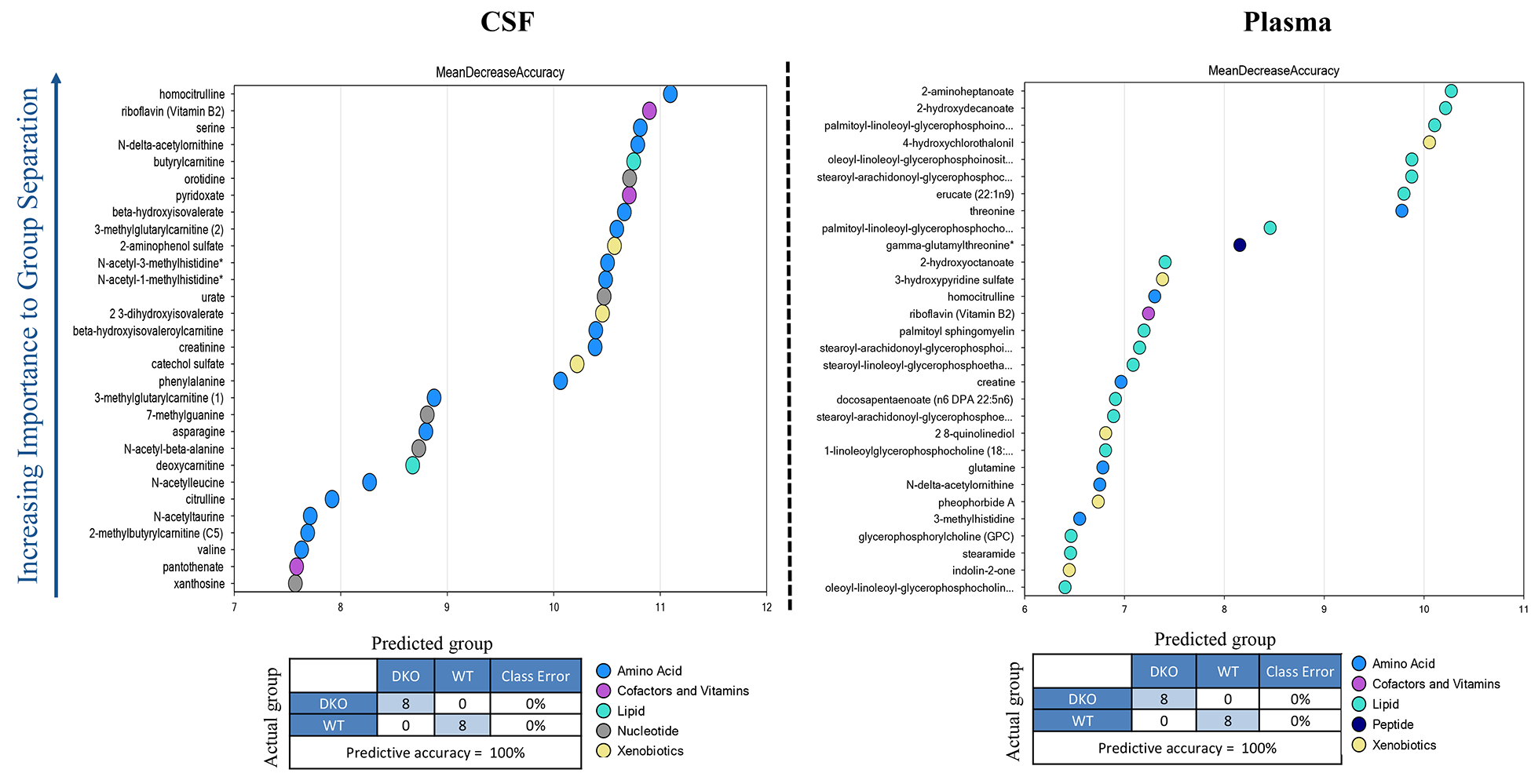

Supplement: S3 Fig — Random forest analysis (RFA) was used to calculate group separation and identify metabolites with highest importance for group separation. RFA identified two groups (WT and KO) with 100% accuracy in both CSF (A) and plasma (B). Metabolites were plotted against their Mean Decrease in Accuracy, calculated by RFA. Metabolites have higher importance on group separation as we move up along the Y-axis and they are also color-coded to identify which pathway the metabolites belong to. (TIF) [file pone.0253852.s003.tif]

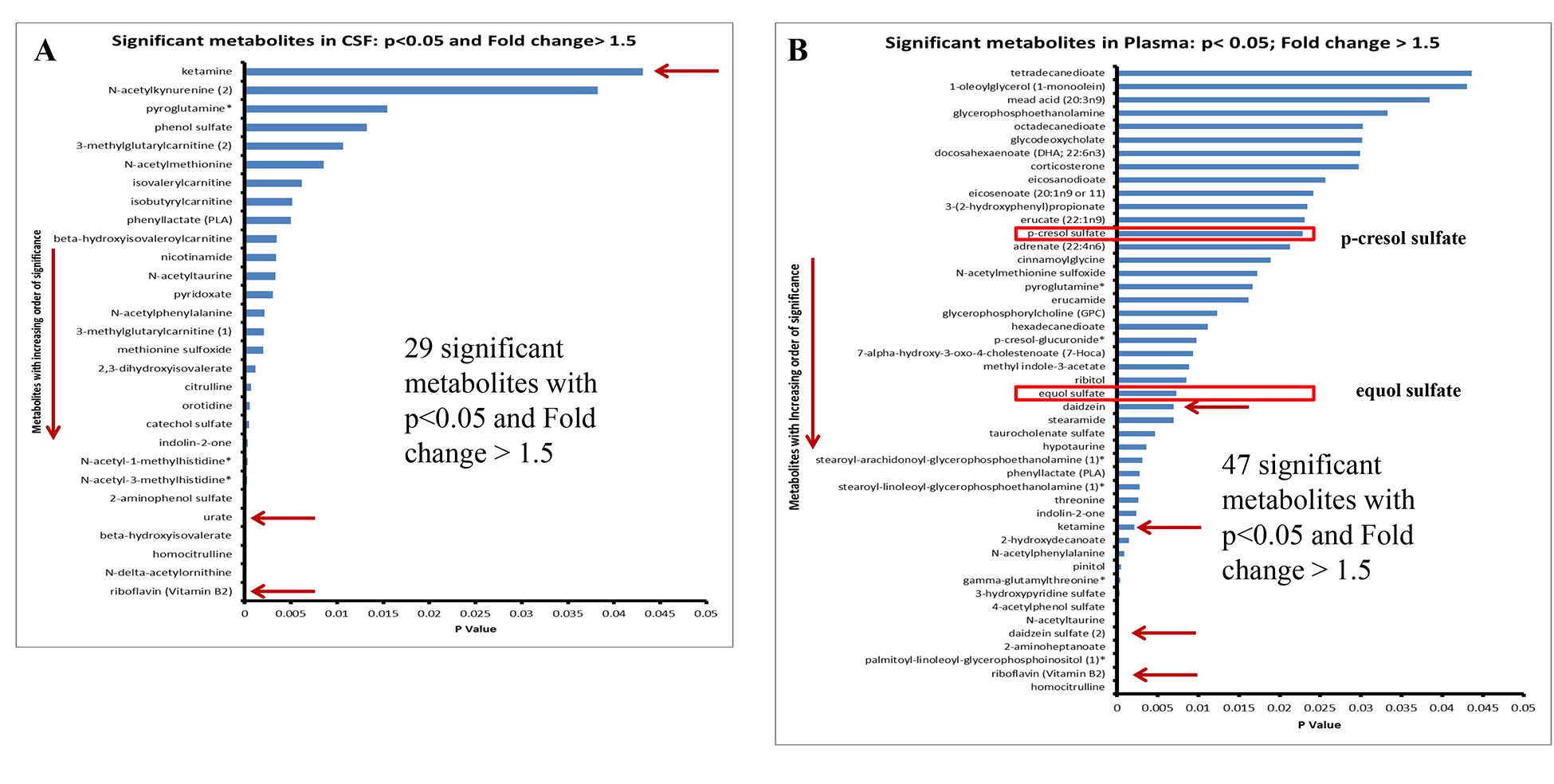

Supplement: S4 Fig — Raw data from Metabolon were analyzed using MetaboAnalyst 3.0. Significant metabolites were identified based on p < 0.05 and fold change > 1.5. Fold change of all significant metabolites were plotted against their p-value. The red arrow indicates known substrates of Bcrp and/or Pgp. (TIF) [file pone.0253852.s004.tif]

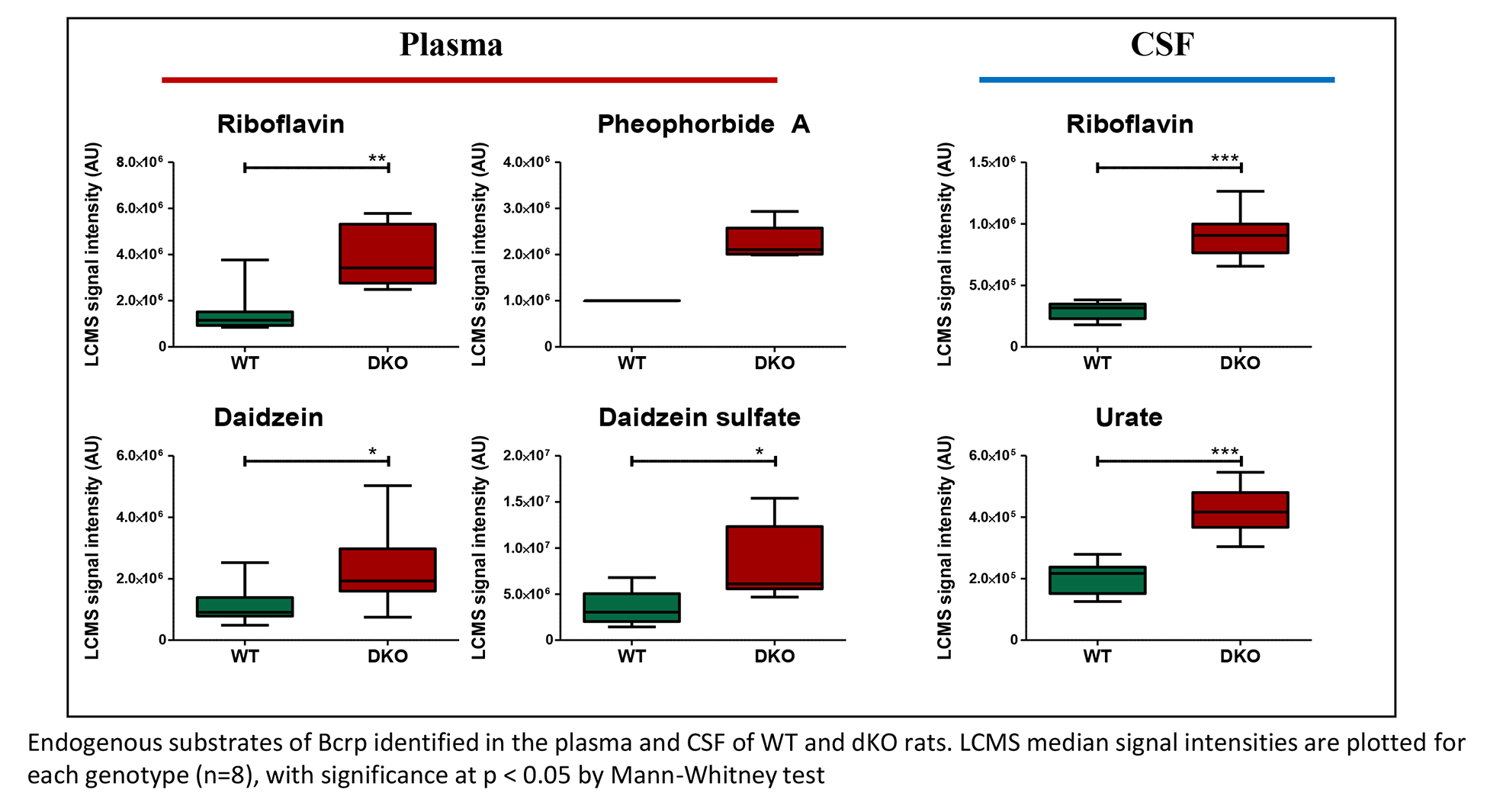

Supplement: S5 Fig — Raw LCMS signal intensities are plotted for each genotype (n = 8), with significance calculated at p < 0.05 by the Mann-Whitney test. (TIF) [file pone.0253852.s005.tif]

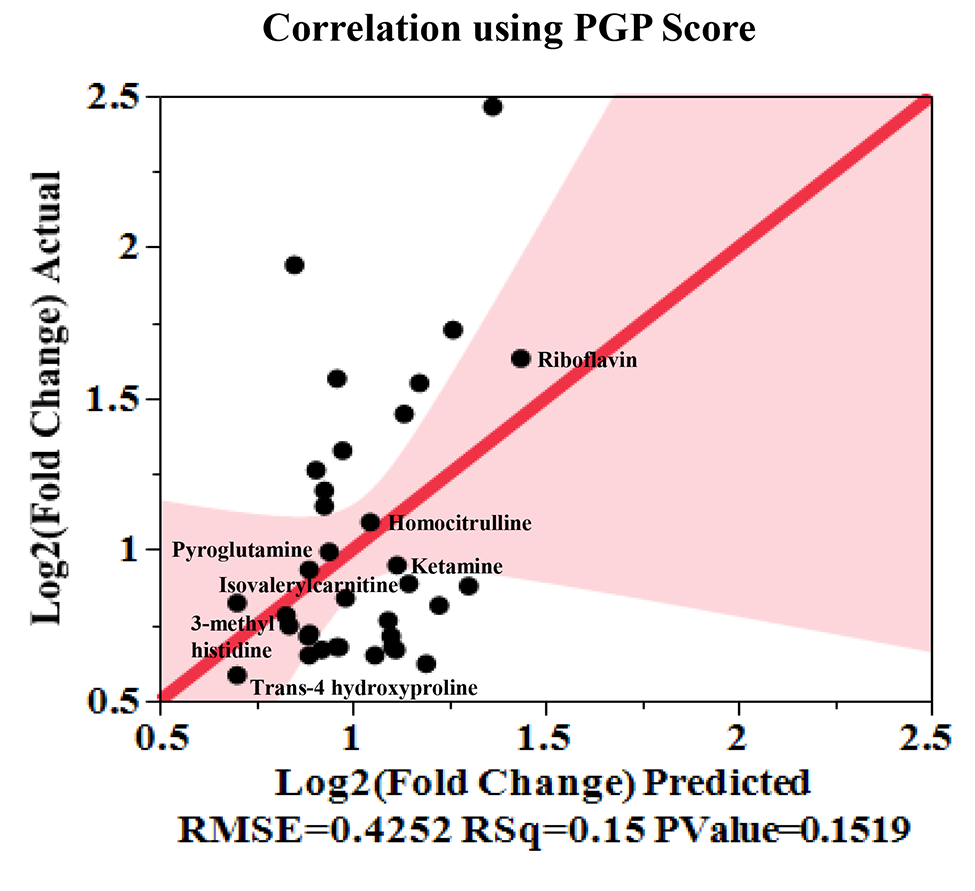

Supplement: S6 Fig — Log 2 (fold change) of significantly altered CSF metabolites with > 1.5 fold increase in dKO rats over WT did not correlate with PGP score, logP and molecular polar surface area of the metabolites (p = 0.1519). (TIF) [file pone.0253852.s006.tif]

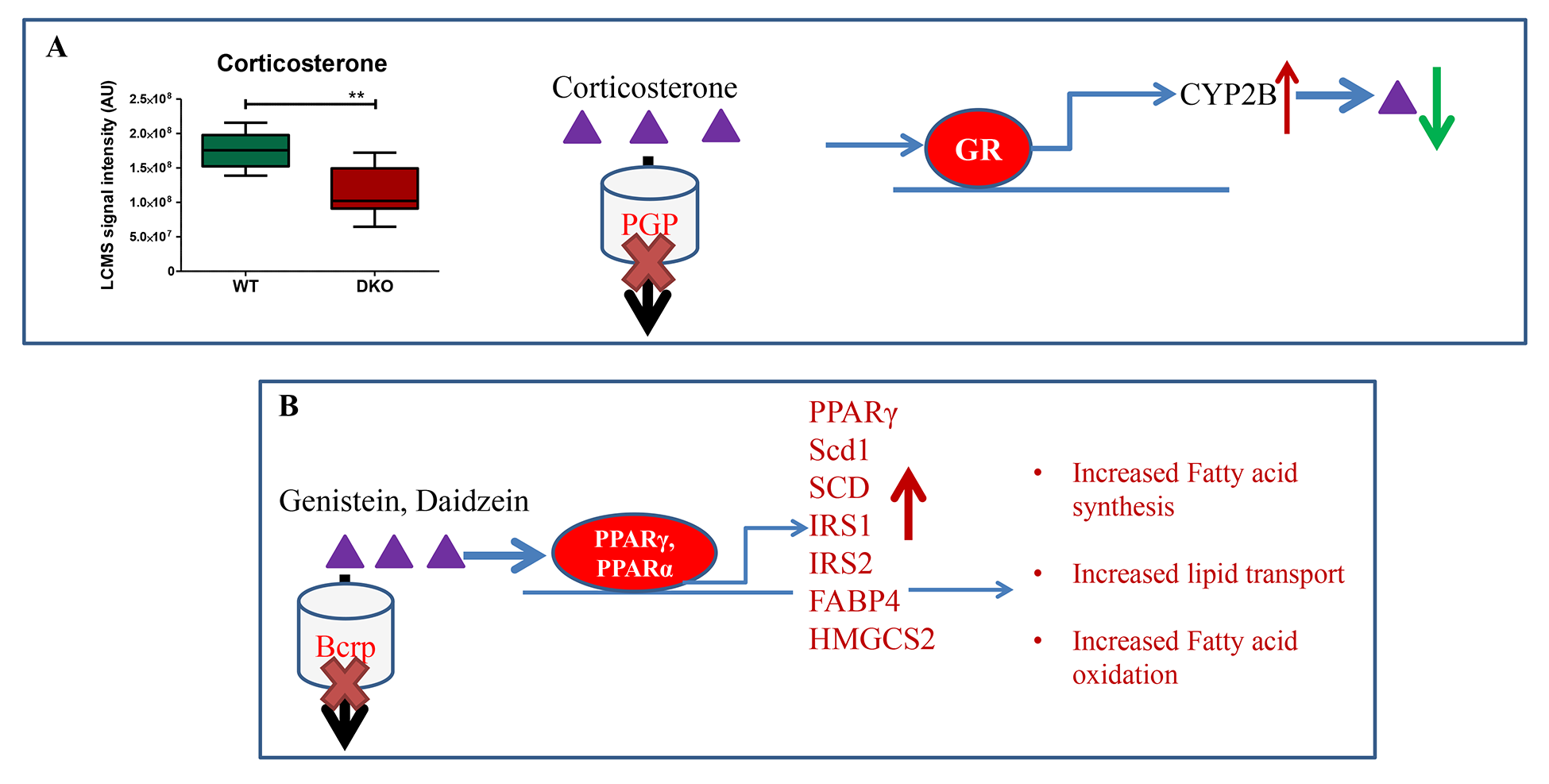

Supplement: S7 Fig — Proposed mechanism for reduction in corticosterone observed in the dKO rat plasma due to glucocorticoid receptor mediated induction of the Cyp2b1 enzyme in dKO rat liver (observed in microarray analysis of liver tissue) (A). Proposed mechanism for reduction of plasma fatty acids by elevated Bcrp substrates genistein and daidzein activating PPARγ (B). (TIF) [file pone.0253852.s007.tif]
